# An Ornithopod-Dominated Tracksite from the Lower Cretaceous Jiaguan Formation (Barremian–Albian) of Qijiang, South-Central China: New Discoveries, Ichnotaxonomy, Preservation and Palaeoecology

**DOI:** 10.1371/journal.pone.0141059

**Published:** 2015-10-22

**Authors:** Lida Xing, Martin G. Lockley, Daniel Marty, Jianping Zhang, Yan Wang, Hendrik Klein, Richard T. McCrea, Lisa G. Buckley, Matteo Belvedere, Octávio Mateus, Gerard D. Gierliński, Laura Piñuela, W. Scott Persons, Fengping Wang, Hao Ran, Hui Dai, Xianming Xie

**Affiliations:** 1 School of Earth Sciences Resources, China University of Geosciences, Beijing 100083, China; 2 Dinosaur Trackers Research Group, University of Colorado at Denver, Colorado, United States of America; 3 Naturhistorisches Museum Basel, Augustinergasse 2, CH-4001 Basel, Switzerland; 4 Institute of Geology and Paleontology, Linyi University, Linyi, Shandong 276000, China; 5 Saurierwelt Paläontologisches Museum, Alte Richt 7, D-92318 Neumarkt, Germany; 6 Peace Region Palaeontology Research Centre, Box 1540, Tumbler Ridge, British Columbia, V0C 2W0, Canada; 7 Museum für Naturkunde, Invalidenstrasse 43, 10115 Berlin, Germany; 8 Departamento de Ciências da Terra (CICEGe-FCT), Universidade Nova de Lisboa, Lisbon 2530−157, Portugal; 9 Moab Giants Tracks Museum, 112 W, SR 313, Moab, Utah, United States of America; 10 Museo del Jurásico de Asturias MUJA (Jurassic Museum of Asturias), Colunga E-33328, Spain; 11 Department of Biological Sciences, University of Alberta 11455 Saskatchewan Drive, Edmonton, Alberta T6G 2E9, Canada; 12 Qijiang District Bureau of Land Resources, Chongqing 401420, China; 13 Key Laboratory of Ecology of Rare and Endangered Species and Environmental Protection, Ministry of Education, Guilin 541004, China; 14 No.208 Hydrogeological and Engineering Geological Team, Chongqing Bureau of Geological and Mineral Resource Exploration and Development, Chongqing 400700, China; University of Oxford, UNITED KINGDOM

## Abstract

The historically-famous Lotus Fortress site, a deep 1.5–3.0-meter-high, 200-meter-long horizonal notch high up in near-vertical sandstone cliffs comprising the Cretaceous Jiaguan Formation, has been known since the 13th Century as an impregnable defensive position. The site is also extraordinary for having multiple tetrapod track-bearing levels, of which the lower two form the floor of part of the notch, and yield very well preserved asseamblages of ornithopod, bird (avian theropod) and pterosaur tracks. Trackway counts indicate that ornithopods dominate (69%) accounting for at least 165 trackmakers, followed by bird (18%), sauropod (10%), and pterosaur (3%). Previous studies designated Lotus Fortress as the type locality of *Caririchnium lotus* and *Wupus agilis* both of which are recognized here as valid ichnotaxa. On the basis of multiple parallel trackways both are interpreted as representing the trackways of gregarious species. *C*. *lotus* is redescribed here in detail and interpreted to indicate two age cohorts representing subadults that were sometimes bipedal and larger quadrupedal adults. Two other previously described dinosaurian ichnospecies, are here reinterpreted as underprints and considered *nomina dubia*. Like a growing number of significant tetrapod tracksites in China the Lotus Fortress site reveals new information about the composition of tetrapod faunas from formations in which the skeletal record is sparse. In particular, the site shows the relatively high abundance of *Caririchium* in a region where saurischian ichnofaunas are often dominant. It is also the only site known to have yielded *Wupus agilis*. In combination with information from other tracksites from the Jiaguan formation and other Cretaceous formations in the region, the track record is proving increasingly impotant as a major source of information on the vertebrate faunas of the region. The Lotus Fortress site has been developed as a spectacular, geologically-, paleontologically- and a culturally-significant destination within Qijiang National Geological Park.

## Introduction

There has long been an absence of Cretaceous dinosaur fossils in south-central China, although the Late Jurassic record is well represented by the rich *Shunosaurus*-*Mamenchisaurus* fauna. Early discoveries of theropod and ornithopod tracks in Lower Cretaceous strata of south-central China offered a small glimpse of the Cretaceous fauna [1], but a more significant ichnological evidence was not reported until after 2007 [2]. Since then, multiple other Lower Cretaceous (Jiaguan Formation) tracksites have been found [3, 4].

Xing et al. [2,4–6] described dinosaur/pterosaur ichnoassemblages from the Lotus tracksite, Qijiang National Geological Park located in Qijiang District, south of Chongqing Municipality near the southeastern border of the Sichuan Basin. The Lotus tracksite includes over 300 tracks of ornithopods, non-avian theropods, birds, pterosaurs and sauropods [6]. Xing et al. [7–10] reported roughly 1000 theropod, sauropod, and ornithopod tracks from the Zhaojue tracksite, Zhaojue Region, near the southern border of the Sichuan Basin. These assemblages helped to fill the gap in the tetrapod fossil record, revealing a distinct change in the ecology of south-central China after the Late Jurassic epoch that was dominated by the *Shunosaurus-Mamenchisaurus* fauna [11].

**Fig 1:**
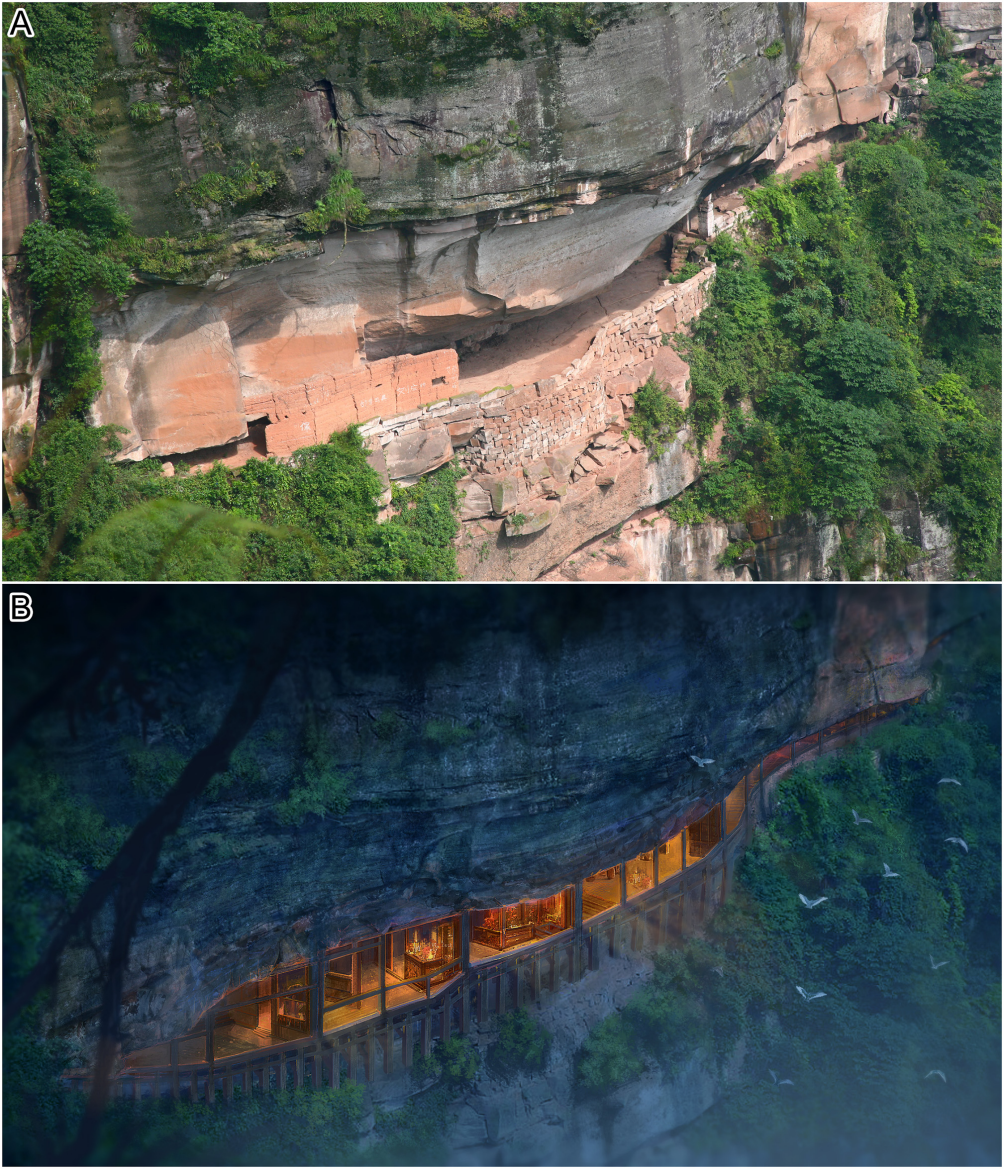
Photograph (A) and proposed future reconstruction (B) of the Lotus tracksite, China. Illustration by Zhongda Chuang.

Because of the well-preserved dinosaur tracks and the unique setting underneath a waterfall in a historic fortress, in the heart of the Danxia landscape, the Lotus tracksite has become a national and international tourist attraction (Fig 1). However, despite the site's fame, many of its fossil tracks have remained poorly described. In November 2012, an international team investigated the Lotus tracksite, mapped the entire site on transparent plastic film (cataloged as CUGB-Q), and measured and photographed selected tracks for 2D and 3D analyses. Here we offer a re-description of these tracks and document new aspects of their morphology, preservation history, and paleoecology.

## History of Research

In 2006, Qijiang Land and Resources Bureau and South-East Sichuan Province Geological Team discovered over 100 dinosaur tracks at the historically-famous Lotus Fortress (GPS: 29° 1'11.62"N, 106°45'26.20"E), Hongyan Village in Laoying Mountain area, Qijiang, Chongqing. Xing et al. [2] described these tracks and attributed them to four vertebrate ichnotaxa: *Caririchnium lotus*, *Wupus agilis*, *Laoyingshanpus torridus* and *Qijiangpus sinensis*. Based in part on the importance of the tracksite, the Ministry of Land and Resources of PRC established Qijiang National Geological Park, in 2009, which includes the track-bearing areas within its protection zone, along with an extensive Jurassic petrified forest.

Xing et al. [12–13] discussed the often surprisingly intimate relationship between dinosaur tracks and Chinese folktales. The name “Lotus” tracksite reflects the local belief that the track site represented lotus leaf veins (the mud cracks) and petals (the ornithopod tracks) submerged in water (the ripple marks). Lotus Fortress is famous as a castle stronghold dating back to the time of the Mongol invasions of the late 13th century (Southern Song Dynasty Baoyou 4th Year, A.D. 1256), and humans have been living at Lotus tracksite for over 700 years (Fig 1). During this period, most tracks were covered with soil to make castle grounds more comfortable and, thus, the tracks were largely protected, despite the abundant human traffic. Today the site, previously very difficult of access, is now approachable by a steep series of about 800 steps designed to help visitors reach the site with relative ease.

In 2011, pterosaur tracks were first recognized by Daqing Li, from the Geological Museum of Gansu, and one of us (FW). In 2012, one 3D *Caririchnium lotus* pes track from the Lotus tracksite was described [5]. Xing et al. [6] gave the first detailed description of the *Pteraichnus* tracks and evaluation of their paleoecological significance. Xing et al. [4] reviewed *Wupus agilis* from the Lotus tracksite. *Wupus*, originally identified as the trace of a small non-avian theropod track-maker [2], is now considered to be the track of a large avian and referred to the ichnofamily Limiavipedidae.

## Geological Setting

### 1 Jiaguan Formation

**Fig 2:**
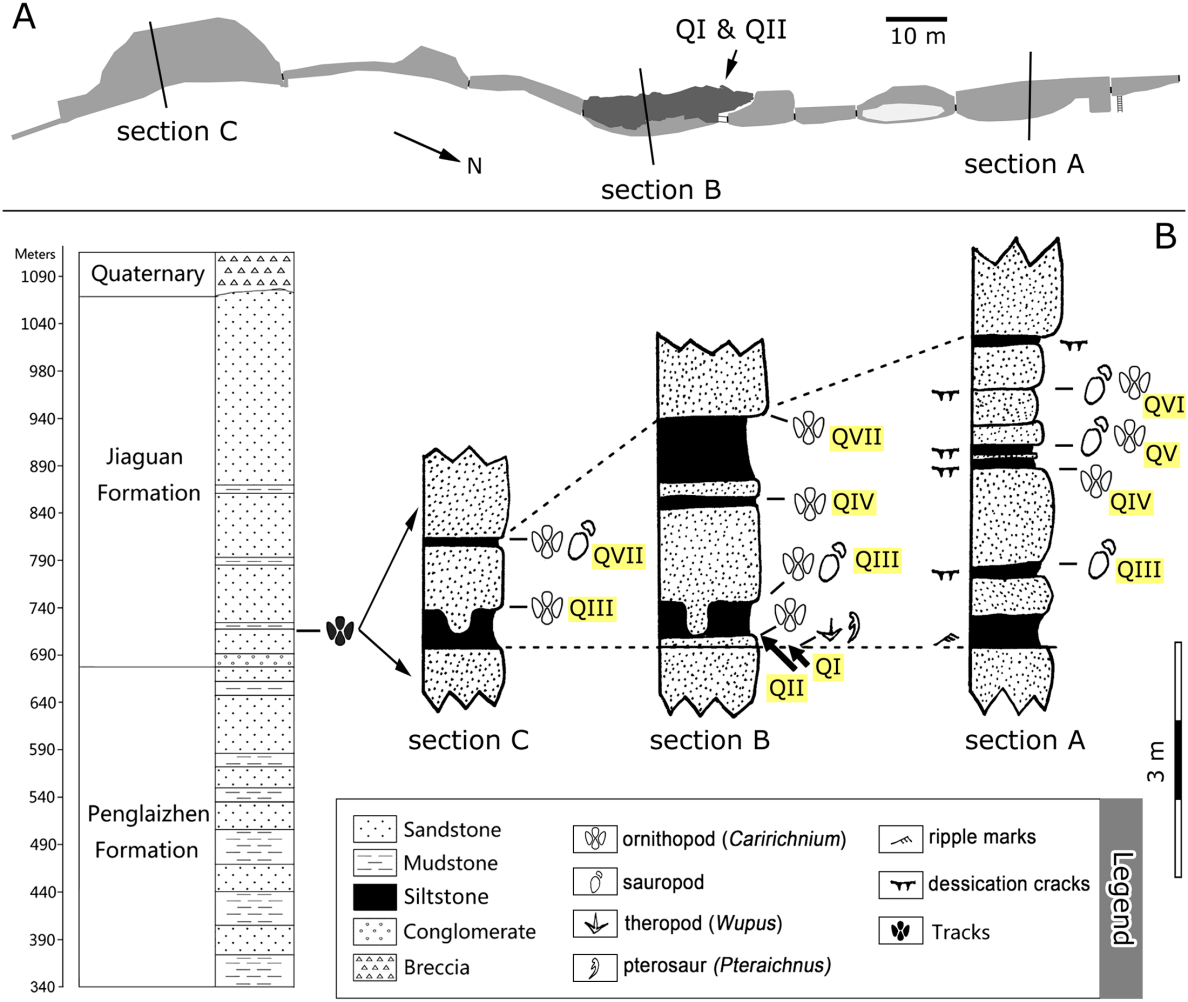
Plan view map of the Lotus tracksite (A) and stratigraphic sections of the Qijiang Lotus tracksite (B).

**Fig 3:**
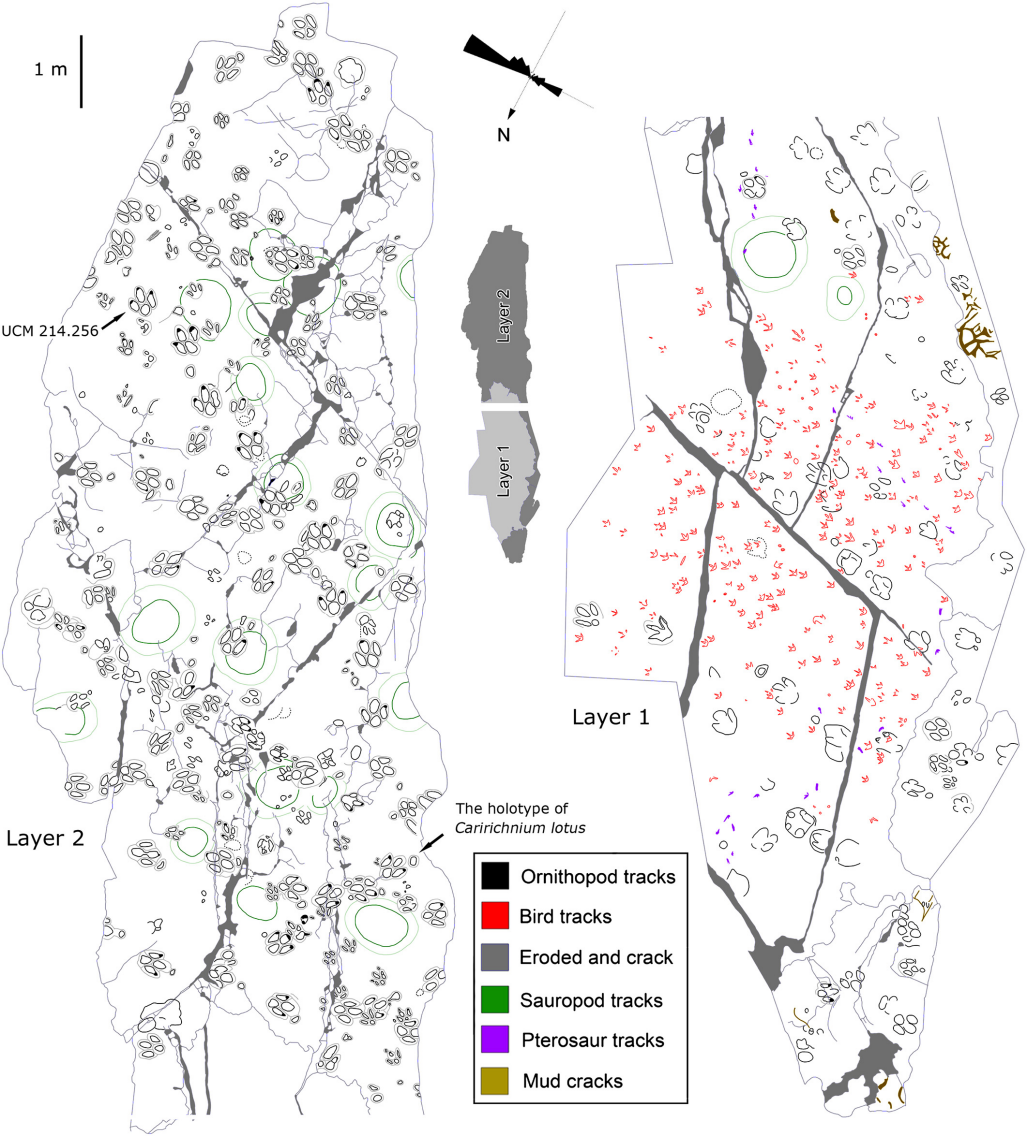
Map of track-bearing levels at QI and II of the Lotus tracksite.

Qijiang National Geological Park is situated in the eastern part of the Yantze Platform and at the southeastern border of Sichuan Basin. From bottom to top, the exposed strata include the Middle Jurassic Shangshaximiao and Suining formations, the Upper Jurassic Penglaizhen Formation, the Lower Cretaceous Jiaguan Formation and unconsolidated Quaternary deposits mainly exposed along river banks and hillsides [14]. The Penglaizhen Formation and the Jiaguan Formation are separated by a non-angular unconformity. Strata at the Lotus tracksite are more than 700 meters thick, with the Upper Jurassic Pengliazhen Formation (about 340 m) at the base and the Lower Cretaceous Jiaguan Formation (about 390 m) on top (Fig 2). The lithological association of the Jiaguan Formation consists of massive sandstones intercalated with thinner mudstone intervals, exposed at the Lotus site in an impressive near-vertical cliff face. The track-and wrinkle structure-bearing levels first occur in dark purple redquartz sandstone in the lower part of the Jiaguan Formation about 30–40 m above the base of the unit [2, 4, 6]. The beds are near-horizontal with the result that notches have been eroded horizontally into the steep vertical cliff faces by removal of the soft siltstones and mudstones. The main tracksite (levels QI and QII) comprise the floor of one of the notches (Figs 1A and 3), developed as the fortress, and reveals tracks that are particularly well preserved. Other track-bearing levels (QIII–QVII) occur within this notch at higher levels (Fig 2).

Based on ostracod distributions, Li et al. [15] referred the lower part of the Jiaguan Formation to the Lower Cretaceous, the middle part to the Middle Cretaceous and the upper part to the Upper Cretaceous. Based on total magnetochronology and ESR dating, the Jiaguan Formation was formed between 117–85 Ma (Aptian–Santonian) [16] and 140–85 Ma (Valanginian–Santonian) [17]. However, recent pollen studies indicate a Barremian–Albian age for the Jiaguan Formation [18] and this latter age assignment is adopted here.

### 2 Depositional environment

At the research area, the Lower Cretaceous Jiaguan Formation in mainly composed of alternating thick purple red sandstone layers and thin purple red mudstone and siltstone layers, and bottom layers of thick conglomerate. The maturity of the Lotus tracksite area sediments is quite high, the rocks largely consist of quartz and feldspar (mainly potassium feldspar) with a little of debris and limestone. The sediments are divided into different zones based on increasing grain sizes from top to bottom. Various bedding plains are present within the purple red sandstone layers, including convolute beddings, tabular cross-bedding, wedge cross-bedding, current bedding, and parallel bedding. Many of the sandstones are lenticular and contain rip-up clasts of the underlying siltstones and mudstones. Some of the sandstone surfaces display current ripples, and deep desiccation cracks are common in the siltstones [6].

Dai et al. [19] analyzed grain size in sandstone samples from the Lotus tracksite and found that the cumulative grain size curve showed a bi-modal pattern that is inferred to represent a moderate slope “bouncing” grain population and low slope suspension population, with the former being dominant. The cut-off points of the bouncing population and the suspension population are between 3 to 3.5Φ. This evidence suggests a meandering river as the likely depositional environment [19].

### 3 Invertebrates traces

The Lotus tracksite also preserves many invertebrate traces, including *Scoyenia gracilis*, *Beaconites antarcticus*, and *Planolites beverleyensis* [19], among which *Scoyenia* dominates. All of these ichnogenera pertain to the *Scoyenia* ichnofacies [20] and are fodinichnia type traces. *Scoyenia* and *Beaconites* reflect intermittent emergence in a low-energy ultra-shallow water environment [20–21]. In river systems, the *Scoyenia* ichnofacies typically appears in over-bank deposits, such as floodplains, ponds, and flood fans [22–23]. *Planolites*, however, is seen in all kinds of sedimentary environments [24]. The trace makers of *Scoyenia* and *Beaconites* were probably arthropods [22, 25]. Buatois and Mangano [26] suggested that the trackmakers of *Planolites* in nonmarine environments were also arthropods.

Invertebrate traces from Emei Region in the western Sichuan Basin also come from the Jiaguan Formation and include at least twelve ichnogenera and two identified ichno-assemblages: (1) *Scoyenia*-*Steinichnus*-*Rusophycus* and (2) *Skolithos*-*Arenicolites*. These traces formed in frequently drought-prone fluvial environments, mostly in flood plain deposit [24, 27]. By studying invertebrate traces in the same area, Chen [28] identified five ichnofabrics: *Arenicolites*, *Skolithos*, *Scoyenia*, *Planolites* and *Palaeophycus*. Invertebrate traces from the Lotus tracksite are similar to those from Emei Region, reflecting a river environment with periodic flooding and frequent droughts.

### 4 Microbial mats

From a macroscopic point of view, Dai et al. [19] identified and described two different wrinkle structure types from the Lotus tracksite. By applying microstructure analysis with a scanning electron microscope (SEM) with energy dispersive spectroscopy (EDS) and other high-resolution instrumentation that detected sheath-like and globular organic matter, Dai et al. [19] inferred the microbial origin of the observed wrinkle structures.

The sandy substrate was covered with a thin (0.5–3 mm) microbial mat with wrinkle structures. When a trackmaker stepped on the microbial mat, a well-defined small displacement rim formed all around the track. The microbial mat served as a water-resisting layer which may have kept underlying sediment relatively moist even while the mat itself was dry. The superficial microbial mat led to cracking around the foot rather than an outward transmission of the applied force; in such case, a relatively deep and well-preserved footprint was formed when a trackmaker walked on this kind of substrate. The presence of a microbial mat appears, therefore, to have been an important, if not the crucial, factor for the exquisite preservation of the vertebrate tracks at the Lotus tracksite. The microbial mat may have enhanced the stabilization and/or early precipitation of carbonate and hence have consolidated the tracks.

## Material and Methods

In November 2012, the entire Lotus site was mapped on transparent plastic film (Fig 3, S1 Fig). The tracks of Layers 1 and 2 were measured and photographed for 2D and 3D analyses. The original tracings on plastic film have been reposited at the Qijiang National Geological Park. Replicas were made of several sets using latex for the initial molds and plaster of Paris and fibreglass replicas. They are housed in the Qijiang National Geological Park Museum, with additional replicas in the University of Colorado collections.

The maximum track length (L), maximum width (W), maximum depth (D), pace length (PL), stride length (SL), pace angulation (PA), rotation (R), trackway width (TW) and the angle between digits II–III, III–IV were measured for the ornithopod trackways.

Photogrammetric images were produced from multiple digital photographs (Canon EOS 5D Mark III) which were converted into scaled, highly accurate 3D textured mesh models using Agisoft Photoscan Professional. The mesh models were then imported into Cloud Compare where the models were rendered with accurately scaled color topographic profiles.

## Distribution of Dinosaur Tracks

**Fig 4:**
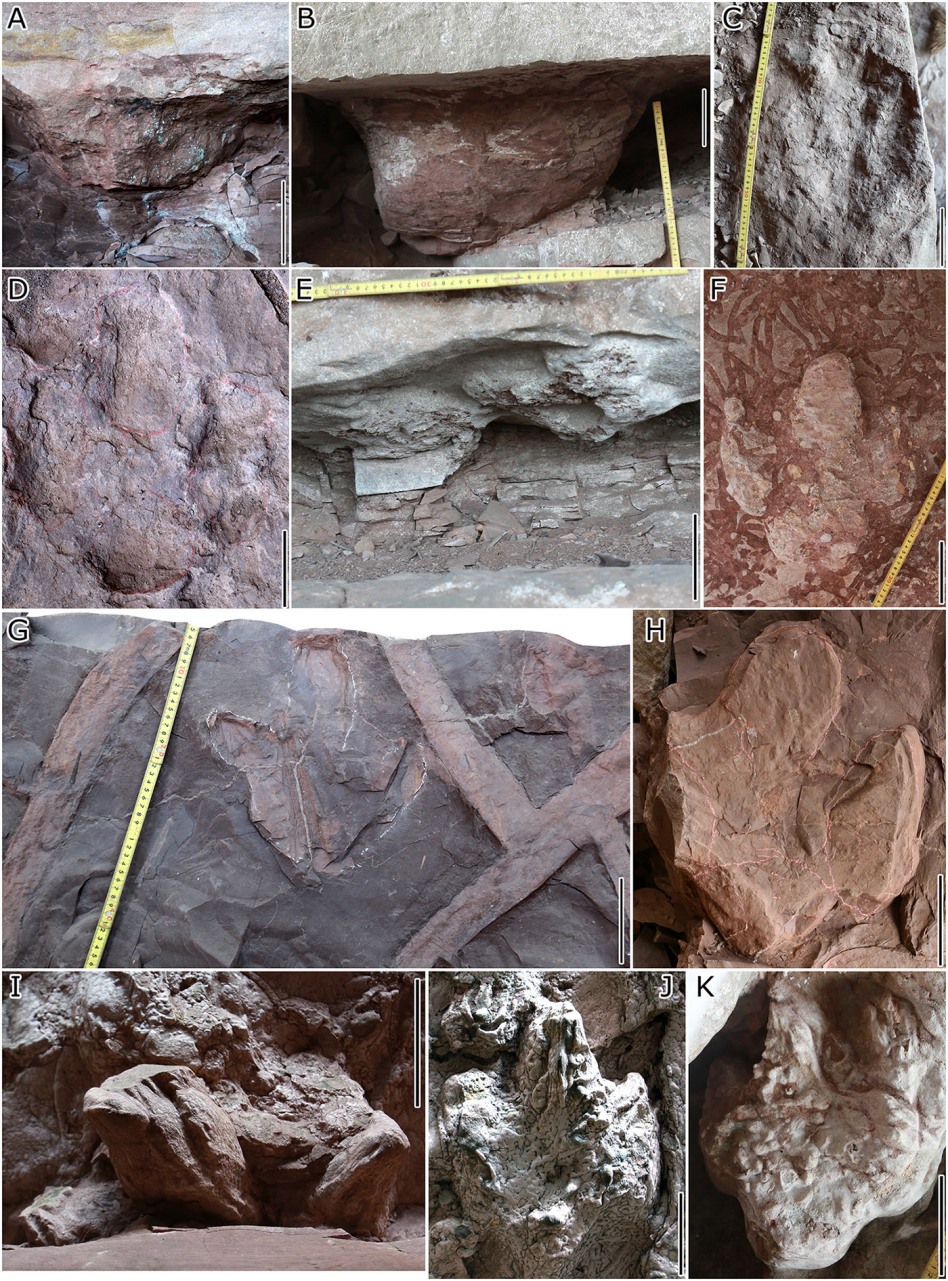
Representative dinosaur tracks from the Lotus tracksite. An ornithopod track cast (A) and sauropod track cast (B) from QIII, ornithopod track mold and cast (C and D respectively) from QIV, ornithopod track casts (E and F) from QV, ornithopod track casts (G and H) from QVI, ornithopod track casts (F, I–K) from QVII.

The tracks occur on at least seven surfaces, referred to as layers QI to QVII (Figs 2 and 4). Due to changes of sediment thickness, we measured three sections (A–C) at the Lotus tracksite. These show a thinning of the section from the NNW (section A) to the SSE (section C).

QI and II: The lowest surface (QI) is dominated by trackways of the small tridactyl ichnospecies *Wupus agilis* and *Pteraichnus* pterosaur trackways. The second layer (QII), is 10 cm higher and contains the trackways of the large ornithopod *Caririchnium lotus* and a few invertebrate traces. These two different ichnoassemblages are present within only a thin stratigraphic interval, and therefore, the depth of the *C*. *lotus* tracks on QII is sufficient to leave undertracks on the QI surface.

QIII: The third layer, about 50 cm above the QII layer. Several infilled tracks are present on the undersides of overlying, bench-forming sandstone layers [5]. The Qijiang District Bureau of Land Resources collected ornithopod casts, including two complexly overprinted series. Additionally, there are seven small ornithopod trackway molds, which most likely belong to *Caririchnium lotus*, on a collapsed sandstone slab.

QIV: The fourth layer is found in both sections B and C and is about 1.2 m above QIII. It preserves mud cracks, ripple marks, and only 2–3 *Caririchnium lotus* molds in poor preservation.

QV and QVI: The fifth and sixth layers are only found in section A and are about 0.5 m and 1m above the fourth layer, respectively. Tracks include both sauropod and ornithopod morphotypes. All sauropod tracks are deep casts. Ornithopod sandstone casts occur with extremely large mud cracks which are regarded as "lotus leafs" by locals [12].

QVII: The seventh layer is identified in sections B and C and is about 1 m higher than the underlying layer. A high density of invertebrate traces co-occurring with dinosaur tracks is a distinguishing feature of this layer. Most of the tracks were left by ornithopods while one is an isolated sauropod track.

## Ornithopod Tracks

### 1 Systematic Ichnology

Ornithischia Seeley, 1888[29]

Ornithopoda Marsh, 1881[30]

Iguanodontipodidae Lockley et al 2014[31]

Ichnogenus *Caririchnium* Leonardi, 1984[32]

Ichnospecies *C*. *lotus*, Xing et al. 2007[2]

#### Holotype

**Fig 5:**
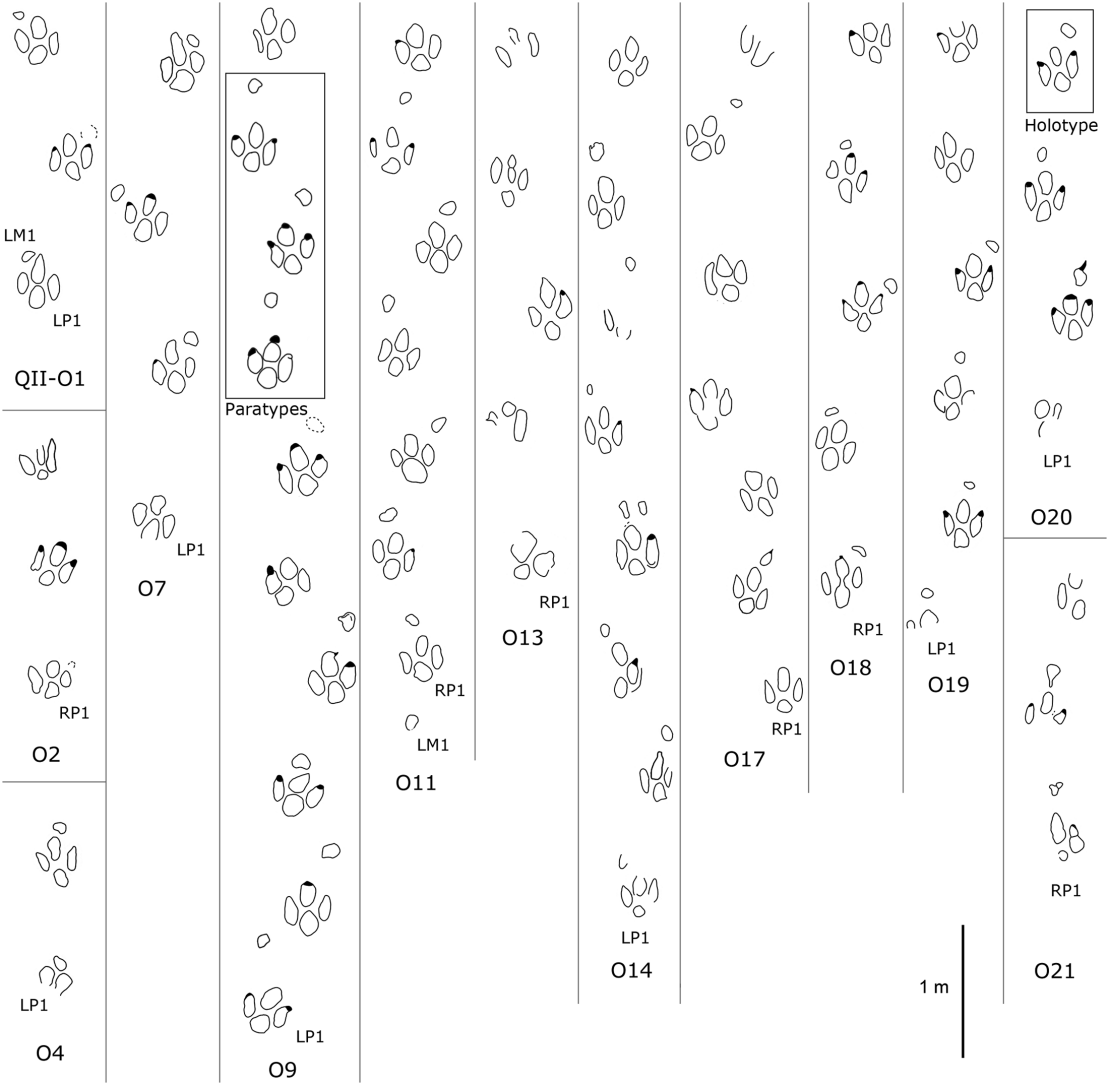
Interpretative outline drawing of large-sized ornithopod trackways from QII, Lotus tracksite, Qijiang, China. Holotype shown in box of trackway QII-O20.

**Fig 6:**
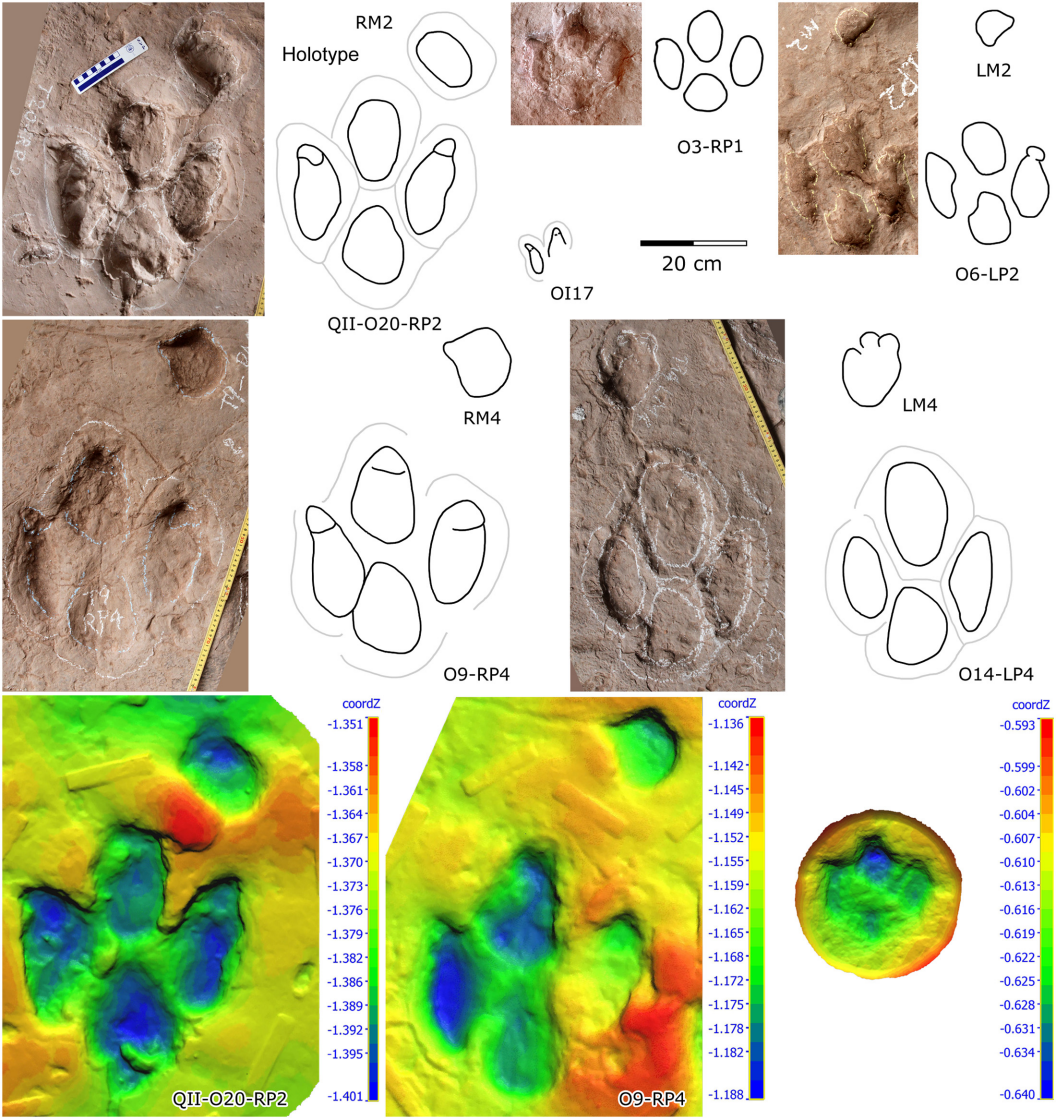
Photographs, interpretative outline drawings and 3D height maps (warm colours are high, cooler colours are low) of well-preserved ornithopod tracks from the Lotus tracksite.

A complete manus-pes set of natural-mold tracks, catalogued as QII-O20-RP2 and RM2 (former specimen number: QJGM-T37-3) from the Lotus tracksite (Figs 5 and 6, Table 1). The original specimens remain in the field.

**Table 1:** Measurements (in cm) of ornithopod tracks from the Lotus tracksite, Chongqing Municipality, China.

Number	L	W	D	R	II-III	III-IV	II-IV	PL	SL	PA	TW	L/W
QII-O1-LP1	41.0	31.5	5.0	-12°	26°	25°	51°	97.0	184.0	—	—	1.3
QII-O1-LM1	5.5	9.5	4.0	—	—	—	—	—	—	—	—	0.6
QII-O1-RP1	37.0	32.5	3.0	—	22°	33°	55°	92.0	—	140°	25.0	1.1
QII-O1-RM1	10.5	10.5	—	—	—	—	—	—	—	—	—	1.0
QII-O1-LP2	39.0	33.0	3.0	-16°	32°	29°	61°	—	—	—	—	1.2
QII-O1-LM2	6.5	8.5	—	—	—	—	—	—	—	—	—	0.8
Mean-P	39.0	32.3	3.7	-14°	27°	29°	56°	94.5	184.0	140°	25.0	1.2
Mean-M	7.5	9.5	4	—	—	—	—	—	—	—	—	0.8
Remarks	Tracks are generally poorly preserved, LP1–LM1 are the best set.											
QII-O2-RP1	28.5	33.5	3.5	—	39	29°	68°	84.0	164.0	—	—	0.9
QII-O2-LP2	34.0	30.5	2.5	—	30°	28°	58°	—	—	—	8.0	1.1
QII-O2-RP2	—	26.5	4.5	—	—	—	—	—	—	—	—	—
Mean-P	31.3	30.2	3.5	—	35°	29°	63°	84.0	164.0	—	8.0	1.0
Remarks	Tracks are generally poorly preserved, attribution of RP2 to trackway is not sure, RP2 poorly preserved with digits being not clearly visible.											
QII-O3-LP1	22.5	19.5	2.0	-30°	32°	27°	59°	77.5	170.0	160°	—	1.2
QII-O3-RP1	18.5	20.0	3.0	—	32°	31°	63°	77.5	—	176°	12.0	0.9
QII-O3-LP2	20.5	18.0	2.0	-12°	29°	34°	63°	—	—	—	—	1.1
QII-O3-RP2	21.0	14.0	—	—	—	—	—	—	—	—	—	1.5
Mean-P	20.6	17.9	2.3	-21°	31°	31°	62°	77.5	170.0	168°	12.0	1.2
Remarks	One of the best preserved trackways. Pes-only trackway. Only in front of LP1 there is some evidence for a poorly preserved manus track.											
QII-O4-LP1	—	—	3.0	—	—	—	—	97.7	—	—	—	—
QII-O4-LM1	—	—	—	—	—	—	—	—	—	—	—	—
QII-O4-RP1	38.8	28.8	1.5	—	28°	27°	55°	—	—	—	—	1.3
QII-O4-RM1	6.0	9.0	3.0	—	—	—	—	—	—	—	—	0.7
Mean-P	38.8	28.8	2.3	—	28°	27°	55°	97.7	—	—	—	1.3
Mean-M	6.0	9.0	3.0	—	—	—	—	—	—	—	—	0.7
Remarks	Only three tracks, all poorly preserved. There is some evidence for a manus track (LM1) in front of LP1, but this is not sure.											
QII-O5-RP1	24.5	26.0	1.5	10°	38°	32°	70°	74.0	148.0	—	—	0.9
QII-O5-RM1	—	—	—	—	—	—		—	—	—	—	—
QII-O5-LP2	25.0	28.0	2.5	7°	33°	35°	68°	79.0	151.0	150°	20.0	0.9
QII-O5-LM2	—	—	—	—	—	—	—	—	—	—	—	—
QII-O5-RP2	27.0	25.0	1.5	4°	22°	35°	57°	78.0	148.0	150°	21.5	1.1
QII-O5-RM2	—	—	—	—	—	—	—	—	—	—	—	—
QII-O5-LP3	23.5	23.5	1.0	0°	37°	30°	67°	74.0	140.0	156°	17.0	1.0
QII-O5-LM3	—	—	—	—	—	—	—	—	—		—	—
QII-O5-RP3	36.0	—	1.5	9°	—	—	—	72.0	139.0	155°	16.0	—
QII-O5-RM3	—	—	—	—	—	—	—	—	—	—	—	—
QII-O5-LP4	26.0	23.0	2.5	20°	33°	29°	62°	71.3	—	—	—	1.1
QII-O5-LM4	7.5	9.5	—	—	—	—	—	—	—	—	—	0.8
QII-O5-RP4	26.5	22.5	3.0	—	20°	31°	51°	—	130	—	—	1.2
QII-O5-RM4	—	—	—	—	—	—	—	—	—	—	—	—
QII-O5-LP5	—	—	—	—	—	—	—	—	—	—	—	—
QII-O5-LM5	—	—	—	—	—	—	—	—	—	—	—	—
QII-O5-RP5	23.5	26.5	—	—	34°	40°	74°	—	—	—	—	0.9
QII-O5-RM5	5.0	7.0	—	—	—	—		—	—	—	—	0.7
Mean-P	26.5	24.9	1.9	8°	31°	30°	63°	74.7	142.7	153°	18.6	1.0
Mean-M	6.3	8.3	—	—	—	—	—	—	—	—	—	0.8
Remarks	LP3 is poorly preserved; RP3 is cracked and the width cannot be measured; length and width of RP4 cannot be measured											
QII-O6-LP1	—	—	—	—	—	—	—	74.5	—	—	—	—
QII-O6-LM1	—	—	—	—	—	—	—	—	—	—	—	—
QII-O6-RP1	27.0	—	2.5	—	26°	35°	61°	74.0	138.0	—	—	—
QII-O6-RM1	4.5	6.0	4.0	—	—	—	—	70.0	—	—	—	0.8
QII-O6-LP2	24.0	21.0	3.0	—	32°	27°	59°	—	—	—	—	1.1
QII-O6-LM2	4.5	7.0	3.0	—	—	—	—	—	—	—	—	0.6
QII-O6-RP2	—	23.5	—	—	—	—	—	—	—	—	—	—
Mean-P	25.5	22.3	2.8	—	29°	31°	60°	74.3	138.0	—	—	1.1
Mean-M	4.5	6.5	3.5	—	—	—	—	70.0	—	—	—	0.7
Remarks	First track LP1 is very poorly preserved. There are only 2 reasonably-well preserved tracks, and for this reason the trackway orientation is not very well defined.											
QII-O7-RP1	38.0	30.0	3.5	-17°	27°	25°	52°	—	228.0	11°	—	1.3
QII-O7-RM1	8.5	12.0	3.0	—	—	—	—	—	—	—	—	0.7
QII-O7-LP2	32.5	30.0	3.5	—	28°	30°	58°	115.0	—	170°	23.0	1.1
QII-O7-LM2	7.0	10.5	3.0	—	—	—	—	—	—	—	—	0.7
QII-O7-RP2	48.0	31.0	4.0	-22°	22°	23°	45°	—	—	—	—	1.5
QII-O7-RM2	5.5	8.0	1.5	—	—	—	—	—	—	—	—	0.7
Mean-P	39.5	30.3	3.7	-20°	26°	26°	52°	115.0	228.0	144°	23.0	1.3
Mean-M	7.0	10.2	2.5	—	—	—	—	—	—	—	—	0.7
Remarks	Manus tracks always close in front of pes, but in a more exterior position.											
QII-O8-RP1	27.0	21.0	1.0	—	33°	31°	64°	61.5	116.1	173°	—	1.3
QII-O8-RM1	—	—	—	—	—	—	—	—	—	—	—	
QII-O8-LP2	23.0	20.5	1.5	—	31°	30°	61°	55.2	117.7	174°	—	1.1
QII-O8-LM2	—	—	—	—	—	—	—	—	—	—	—	
QII-O8-RP2	22.0	20.0	1.5	—	37°	30°	67°	63.2	—	—	—	1.1
QII-O8-RM2	2.5	4.5	—	—	—	—	—	67.4	—	—	—	0.6
QII-O8-LP3	(22)	(18)	1.0	—	30°	33°	63°	—	—	—	—	
QII-O8-LM3	3.0	6.0	—	—	—	—	—	—	—	—	—	0.5
QII-O8-RP3	(23)	—	2.0	—	—	—	—	—	—	—	—	—
Mean-P	24.0	20.5	1.4	—	33°	31°	64°	60.0	116.9	174°	—	1.2
Mean-M	2.8	5.3	—	—	—	—	—	67.4	—	—	—	0.6
Remarks	Each of digit traces of these tracks are more slender than others.											
QII-O9-LP1	37.0	32.0	2.5	18°	28°	40°	68°	85.0	166.0	—	—	1.2
QII-O9-LM1	7.0	11.0	2.5	—	—	—	—	84.1	137.0	125°	—	0.6
QII-O9-RP1	39.0	34.0	2.0	-7°	26°	26°	52°	87.0	171.0	160°	22.0	1.1
QII-O9-RM1	9.0	11.0	3.0	19°	—	—	—	71.0	172.0	144°	—	0.8
QII-O9-LP2	33.5	36.0	3.5	-18°	22°	35°	57°	88.0	158.0	160°	16.0	0.9
QII-O9-LM2	7.5	11.0	2.0	—	—	—	—	109.7	—	—	—	0.7
QII-O9-RP2	39.0	34.5	2.5	-6°	27°	30°	57°	80.0	156.0	123°	29.0	1.1
QII-O9-RM2	8.5	13.0	3.0	—	—	—	—	—	149.2	—	—	0.7
QII-O9-LP3	35.0	—	2.0	-11°	23°	27°	49°	83.0	161.0	160°	22.0	—
QII-O9-LM3	—	—	—	—	—	—	—	—	—	—	—	—
QII-O9-RP3	37.0	34.5	3.5	-12°	30°	32°	62°	82.0	166.0	122°	16.0	1.1
QII-O9-RM3	—	—	—	—	—	—	—	98.6	170.2	143°	—	
QII-O9-LP4	34.0	32.0	3.0	-12°	25°	30°	55°	88.0	164.0	127°	18.0	1.1
QII-O9-LM4	7.0	10.0	3.0	75°	—	—	—	81.0	161.0	138°	—	0.7
QII-O9-RP4	39.0	33.0	2.5	-8°	30°	29°	59°	80.0	159.0	150°	18.0	1.2
QII-O9-RM4	8.0	10.0	2.0	—	—	—	—	92.0	—	—	—	0.8
QII-O9-LP5	38.5	30.0	—	—	29°	32°	61°	82.0	—	160°	—	1.3
QII-O9-LM5	7.5	10.0	2.5	—	—	—	—	—	—	—	—	0.8
QII-O9-RP5	36.0	—	2.5	-19°	30°	25°	55°	—	—	—	—	—
QII-O9-RM5	—	—	—	—	—	—	—	—	—	—	—	—
Mean-P	36.8	33.3	2.7	|12°|	27°	31°	58°	83.9	162.6	145°	20.1	1.1
Mean-M	7.8	10.9	2.6	47°	—	—	—	89.4	157.9	138°	—	0.7
Remarks	Best preserved trackway. Pes and manus tracks are always present unless that they are located in a "fault zone". Some missing manus tracks may also be overprinted by pes tracks. RM1 with impression of? digits; LP3 partially overprinted by T13-RP3; LM3 not preserved-possibly in normal fault; RM3 is overprinted by track of another trackway; RP4 is the second best preserved pes; RM4 is the best preserved manus-cast; LP5-LM5 is best preserved pes-manus cast by M. Lockley.											
QII-O10-LP1	27.0	25.0	2.0	—	30°	30°	60°	69.3	142.8	157°	—	1.1
QII-O10-LM1	4.0	6.0	0.5	—	—	—	—	—	137.3	—	—	0.7
QII-O10-RP1	(20)	(20)	2.0	—	—	—	—	76.9	142.2	146°	—	—
QII-O10-RM1	—	—	—	—	—	—	—	—	—	—	—	—
QII-O10-LP2	(23)	24.0	2.0	—	30°	36°	66°	72.9	—	—	—	—
QII-O10-LM2	3.0	7.0	0.5	—	—	—	—	73.3	—	—	—	0.4
QII-O10-RP2	(30)	22.0	2.0	—	24°	23°	47°	—	128.4	—	—	—
QII-O10-RM2	5.5	6.6	0.5	—	—	—	—	—	134.8	—	—	0.8
QII-O10-LP3	—	—	—	—	—	—	—	—	—	—	—	—
QII-O10-LM3	(4)	(8)	0.5	—	—	—	—	—	—	—	—	—
QII-O10-RP3	28.0	22.0	1.0	—	35°	27°	62°	—	—	—	—	1.3
QII-O10-RM3	3.3	6.6	1.0	—	—	—	—	—	—	—	—	0.5
Mean-P	27.5	23.3	1.8	—	30°	29°	59°	73.0	137.8	152°	—	1.2
Mean-M	2.4	3.6	0.6	—	—	—	—	73.3	136.1	—	—	0.6
Remarks	No well-preserved left pes and manus, only two right pes-manus pairs.											
QII-O11-LM1	7.5	10.0	2.0	54°	—	—	—	71.7	146.0	166°	—	0.8
QII-O11-RP1	34.0	31.0	3.0	0°	37°	23°	60°	83.0	154.5	—	—	1.1
QII-O11-RM1	6.5	10.0	2.0	33°	—	—	—	74.6	137.5	139°	—	0.7
QII-O11-LP2	33.5	30.0	3.0	-10°	29°	25°	54°	76.0	152.0	160°	17.5	1.1
QII-O11-LM2	7.5	12.0	2.0	29°	—	—	—	72.3	148.5	130°	—	0.6
QII-O11-RP2	35.5	32.0	2.0	-15°	30°	26°	56°	81.0	159.0	164°	12.5	1.1
QII-O11-RM2	8.0	12.0	0.5	-45°	—	—	—	92.6	151.3	126°	—	0.7
QII-O11-LP3	34.5	30.0	2.0	-12°	31°	25°	56°	83.0	153.0	154°	20.0	1.2
QII-O11-LM3	6.5	10.0	2.0	55°	—	—	—	78.5	144.0	127°	—	0.7
QII-O11-RP3	36.5	31.5	2.0	0°	22°	30°	52°	89.0	149.0	142°	27.0	1.2
QII-O11-RM3	8.0	11.0	1.5	—	—	—	—	82.2	—	—	—	0.7
QII-O11-LP4	36.0	33.0	2.5	-6°	35°	28°	63°	79.0	—	142°	25.5	1.1
QII-O11-LM4	7.0	10.0	2.0	—	—	—	—	—	—	—	—	0.7
QII-O11-RP4	35.0	32.0	2.5	-3°	31°	31°	62°	—	—	—	—	1.1
Mean-P	35.0	31.4	2.4	|6.6°|	31°	27°	58°	81.8	153.5	152°	20.5	1.1
Mean-M	7.3	10.7	1.7	|43°|	—	—	—	78.7	145.5	138°	—	0.7
Remarks	Except of RP4, all pes with manus prints.											
QII-O12-RP1	—	—	—	—	—	—	—	58.8	122.3	154°	—	—
QII-O12-RM1	—	—	—	—	—	—	—	—	—	—	—	—
QII-O12-LP2	20.5	20.0	0.5	—	34°	30°	64°	66.4	125.0	160°	—	1.0
QII-O12-LM2	3.0	6.0	0.5	—	—	—	—	69.0	120.6	154°	—	0.5
QII-O12-RP2	22.0	19.5	1.0	—	36°	28°	64°	60.5	—	—	—	1.1
QII-O12-RM2	5.0	5.0	0.5	—	—	—	—	58.8	—	—	—	1.0
QII-O12-LP3	20.0	17.0	1.0	—	37°	35°	72°	—	—	—	—	1.2
QII-O12-LM3	2.0	6.0	0.5	—	—	—	—	—	—	—	—	0.3
Mean-P	20.8	18.8	0.8	—	36°	31°	67°	61.9	123.7	157°	—	1.1
Mean-M	3.3	5.7	0.5	—	—	—	—	63.9	120.6	154°	—	0.6
Remarks	Only the RP2-RM2 pair is well-preserved.											
QII-O13-RP1	(35)	—	3.0	—	—	—	—	—	—	—	—	—
QII-O13-LP2	(30)	(31)	2.0	—	—	—	—	—	—	—	—	
QII-O13-RP2	44.0	33.0	2.0	-2°	29°	19°	48°	88.0	175.0	167°	—	1.3
QII-O13-LP3	39.0	29.0	2.0	—	22°	20°	42°	88.3	—	—	—	1.3
QII-O13-RP3	36.0	31.0	2.0	—	24°	22°	45°	—	—	—	—	1.2
Mean-P	39.7	31.0	2.2	-2°	25°	20°	45°	88.2	175.0	167°	—	1.3
Remarks	Only pes tracks preserved; no evidence for manus tracks.											
QII-O14-LP1	32.0	29.0	2.5	-6°	20°	29°	49°	92.0	174.0	—	—	1.1
QII-O14-LM1	6.0	11.0	1.5	—	—	—	—	94.0	162.0	130°	—	0.5
QII-O14-RP1	37.0	28.0	3.0	8°	29°	16°	45°	87.0	166.0	160°	17.5	1.3
QII-O14-RM1	7.0	10.0	2.5	—	—	—	—	84.8	158.7	132°	—	0.7
QII-O14-LP2	37.0	26.0	3.0	0°	—	—	—	86.0	173.0	154°	20.5	1.4
QII-O14-LM2	7.0	11.5	2.0	—	—	—	—	90.5	169.0	139°	—	0.6
QII-O14-RP2	34.0	30.0	3.5	12°	24°	27°	51°	92.0	174.0	160°	17.0	1.1
QII-O14-RM2	5.5	9.5	2.0	—	—	—	—	91.5	171.3	138°	—	0.6
QII-O14-LP3	34.0	27.0	2.0	-8°	25°	25°	50°	97.0	165.0	156°	19.0	1.3
QII-O14-LM3	5.5	10.0	2.0	—	—	—	—	93.0	166.0	145°	—	0.6
QII-O14-RP3	—	—	2.0	—	—	—	—	74.0	182.0	156°	15.0	
QII-O14-RM3	7.0	10.0	1.5	—	—	—	—	82.0	—	—	—	0.7
QII-O14-LP4	37.0	28.0	2.0	-5°	30°	24°	53°	104.0	—	160°	14.5	1.3
QII-O14-LM4	6.5	11.0	2.5	—	—	—	—	—	—	—	—	0.6
QII-O14-RP4	37.0	30.0	3.0	-10°	22°	30°	52°	—	—	—	—	1.2
Mean-P	35.4	28.3	2.6	|7°|	25°	25°	50°	90.3	172.3	158°	17.3	1.2
Mean-M	6.4	10.4	2.0	—	—	—	—	89.3	165.4	137°	—	0.6
Remarks	RP2 is located within a sauropod undertrack; LM1 is cut by a normal fault; RP3 is incomplete-except the rear it to overprinted by O9-RP2.											
QII-O15-LP1	21.0	21.0	2.0	-10°	—	32°	—	62.0	131.0	—	—	1.0
QII-O15-LM1	3.0	3.0	0.5	—	—	—	—	55.1	—	—	—	1.0
QII-O15-RP1	24.0	20.0	2.0	-5°	34°	37°	71°	72.0	135.0	170°	18.0	1.2
QII-O15-RM1	5.0	8.0	1.5	36°	—	—	—	—	132.0	—	—	0.6
QII-O15-LP2	31.0	28.0	2.0	-10°	28°	34°	62°	67.0	130.0	150°	17.0	1.1
QII-O15-LM2	5.0	10.0	0.5	—	—	—	—	—	—	—	—	0.5
QII-O15-RP2	25.0	22.0	2.0	-10°	29°	33°	62°	69.0	135.0	150°	17.0	1.1
QII-O15-RM2	5.0	7.0	1.0	57°	—	—	—	65.6	—	—	—	0.7
QII-O15-LP3	27.0	21.0	2.0	-10°	23°	22°	45°	72.0	122.0	150°	14.0	1.3
QII-O15-LM3	6.0	6.0	1.0	—	—	—	—	—	—	—	—	1.0
QII-O15-RP3	22.5	20.0	2.0	-20°	27°	27°	54°	58.0	112.0	150°	17.0	1.1
QII-O15-RM3	4.0	7.0	1.0	—	—	—	—	—	—	—	—	0.6
QII-O15-LP4	26.0	19.0	2.0	-10°	17°	51°	68°	68.0	131.0	120°	26.0	1.4
QII-O15-LM4	—	—	—	—	—	—	—	—	—	—	—	—
QII-O15-RP4	30.0	25.0	1.5	-20°	29°	36°	65°	76.0	—	120°	30.0	1.2
QII-O15-RM4	—	—	—	—	—	—	—	—	—	—	—	—
QII-O15-LP5	26.0	24.0	1.5	-15°	34°	28°	62°	—	—	—	—	1.1
QII-O15-LM5	6.0	8.0	1.5	—	—	—	—	—	—	—	—	0.8
Mean-P	25.8	22.2	1.9	|12°|	28°	33°	61°	68.0	128.0	144°	19.9	1.2
Mean-M	4.9	7.0	1.0	47°	—	—	—	60.4	132.0	—	—	0.7
Remarks	RP2-RM2 pair, and RP4 traces are well-preserved.											
QII-O16-LP1	23.0	18.0	0.8	—	—	—	—	49.2	109.5	168°	—	1.3
QII-O16-RP1	20.0	—	0.9	—	—	—	—	60.5	—	—	—	
QII-O16-LP2	21.5	19.0	1.2	—	—	—	—	—	—	—	—	1.1
Mean-P	21.5	18.5	1.0					54.9	109.5	168°		1.2
Remarks	LM1, RM1 and LM2 are missing											
QII-O17-RP1	30.3	28.0	3.0	-10°	30°	25°	55°	79.0	151.0	—	—	1.1
QII-O17-RM1	—	—	—	—	—	—	—	—	—	—	—	—
QII-O17-LP2	30.2	29.0	3.0	-30°	28°	26°	54°	76.0	140.0	165°	16.0	1.0
QII-O17-LM2	10.0	7.0	1.5	—	—	—	—	—	—	—	—	1.4
QII-O17-RP2	36.0	29.0	28.0	-19°	20°	29°	49°	74.0	158.0	166°	23.0	1.2
QII-O17-RM2	—	—	—	—	—	—	—	—	—	—	—	—
QII-O17-LP3	37.0	—	1.7	-8°	22°	26°	48°	94.0	196.0	145°	28.0	—
QII-O17-LM3	—	—	—	—	—	—	—	—	—	—	—	—
QII-O17-RP3	35.0	33.5	2.5	-23°	14°	28°	42°	106.0	17.0	160°	16.0	1.0
QII-O17-RM3	11.0	6.0	2.3	—	—	—	—	—	—	—	—	1.8
QII-O17-LP4	36.0	32.0	2.9	-15°	24°	33°	57°	82.0	—	136°	29.0	1.1
QII-O17-LM4	10.0	12.0	1.4	—	—	—	—	—	—	—	—	0.8
QII-O17-RP4	—	—	3.3	—	—	—	—	—	—	—	—	—
QII-O17-RM4	—	—	—	—	—	—	—	—	—	—	—	—
Mean-P	34.1	30.3	6.3	|18°|	23°	28°	51°	85.2	132.4	154°	22.4	1.1
Mean-M	10.3	8.3	1.7	—	—	—	—	—	—	—	—	1.3
Remarks	The rear of RP1 is incomplete. LM1, RM1, LM3 and RM4 are all missing. RP4 is only partially preserved.											
QII-O18-LM1	4.0	11.0	2.5	—	—	—	—	—	—	—	—	0.4
QII-O18-RP1	40.0	33.0	3.0	-5°	26°	23°	49°	102.0	202.0	—	—	1.2
QII-O18-RM1	4.0	9.0	0.5	37°	—	—	—	110.0	207.0	143°	—	0.4
QII-O18-LP2	39.0	31.0	3.0	-18°	30°	20°	50°	102.0	197.0	170°	13.0	1.3
QII-O18-LM2	7.0	10.0	2.5	21°	—	—	—	108.8	207.8	137°	—	0.7
QII-O18-RP2	39.0	30.0	2.5	-9°	30°	31°	61°	100.0	197.0	162°	16.0	1.3
QII-O18-RM2	7.0	10.0	1.5	—	—	—	—	—	—	—	—	0.7
QII-O18-LP3	39.0	35.0	4.0	-8°	26°	27°	53°	100.0	—	165°	14.0	1.1
QII-O18-LM3	—	—	—	—	—	—	—	—	—	—	—	—
QII-O18-RP3	36.0	30.0	3.0	-25°	26°	29°	55°	—	—	—	—	1.2
Mean-P	38.6	31.8	3.1	-13°	28°	26°	54°	101.0	198.7	166°	14.3	1.2
Mean-M	5.5	10.0	1.8	29°	—	—	—	109.4	207.4	140°	—	0.6
Remarks	LP2-LM2, RP2-RM2 pairs are well-preserved.											
QII-O19-LP1	—	—	3.0	—	—	—	—	87.0	176.0	—	—	—
QII-O19-LM1	7.5	10.0	1.7	—	—	—	—	87.0	177.8	153°	—	0.8
QII-O19-RP1	30.4	29.5	2.9	0°	29°	29°	58°	94.0	186.0	160°	19.0	1.0
QII-O19-RM1	7.3	10.8	1.7	14°	—	—	—	95.7	179.0	160°	—	0.7
QII-O19-LP2	30.4	28.3	2.4	-10°	31°	31°	62°	92.0	180.0	172°	10.0	1.1
QII-O19-LM2	8.8	8.3	2.0	7°	—	—	—	87.0	—	—	—	1.1
QII-O19-RP2	38.5	29.5	2.4	0°	26°	30°	56°	90.0	177.0	164°	17.0	1.3
QII-O19-RM2	5.7	11.0	1.2	—	—	—	—	—	—	—	—	0.5
QII-O19-LP3	37.5	30.5	2.9	-15°	38°	28°	56°	88.0	—	166°	10.0	1.2
QII-O19-LM3	—	—	—	—	—	—	—	—	—	—	—	—
QII-O19-RP3	30.0	29.5	2.5	-10°	32°	24°	56°	—	—	—	—	1.0
QII-O19-RM3	—	—	—	—	—	—	—	—	—	—	—	—
Mean-P	33.4	29.5	2.7	|7°|	31°	28°	58°	90.2	179.8	166°	14.0	1.1
Mean-M	7.3	10.0	1.7	11°	—	—	—	89.9	178.4	157°	—	0.8
Remarks	LP1 is incomplete and cut by a normal fault, RM4 is missing.											
QII-O20-LP1	—	—	—	—	—	—	—	—	—	—	—	—
QII-O20-LM1	—	—	—	—	—	—	—	105	195.5	146°	—	—
QII-O20-RP1	35.0	33.0	2.0	-5°	30°	28°	58°	91.0	185.0	—	—	1.1
QII-O20-RM1	8.8	13.2	1.5	84°	—	—	—	99.5	189.6	151°	—	0.7
QII-O20-LP2	38.0	30.5	2.5	—	29°	24°	53°	96.0	—	165°	16.0	1.2
QII-O20-LM2	7.4	11.3	3.0	-8°	—	—	—	97.4	—	—	—	0.7
QII-O20-RP2	35.0	30.2	2.5	—	28°	29°	57°	—	—	—	—	1.2
QII-O20-RM2	7.7	11.8	2.7	—	—	—	—	—	—	—	—	0.7
Mean-P	36.0	31.2	2.3	-5°	29°	27°	56°	93.5	185.0	165°	16.0	1.2
Mean-M	8.0	12.1	2.4	|46°|	—	—	—	100.6	192.6	149°	—	0.7
Remarks	LP1 is almost completely overprinted and no measurements are therefore possible. RP2–RM2 pair is the holotype of *Caririchnium lotus*.											
QII-O21-RP1	36.4	—	1.5	-13°	—	25°	—	100.0	190.0	—	—	—
QII-O21-RM1	—	—	—	—	—	—	—	89.0	—	—	—	
QII-O21-LP2	35.5	39.0	2.4	—	—	—	—	94.0	—	165°	21.0	0.9
QII-O21-LM2	—	10.8	1.8	—	—	—	—	—	—	—	—	
QII-O21-RP2	37.7	—	1.7	-8°	—	—	—	—	—	—	—	—
QII-O21-RM2	—	—	—	—	—	—	—	—	—	—	—	—
Mean-P	36.5	39.0	1.9	-11°	—	25°	—	97.0	190.0	165°	21.0	0.9
Mean-M	—	10.8	1.8	—	—	—	—	89	—	—	—	—
Remarks	RM2 is missing.											
QII-O22-RP1	—	—	—	—	—	—	—	—	—	—	—	—
QII-O22-RM1	—	—	—	—	—	—	—	—	—	—	—	—
QII-O22-LP2	19.7	20.0	1.9	-3°	35°	41°	76°	62.0	115.0	—	—	1.0
QII-O22-LM2	4.2	2.4	0.3	—	—	—	—	—	—	—	—	1.8
QII-O22-RP2	21.5	21.2	1.2	-15°	31°	33°	64°	63.0	103.0	170°	7.0	1.0
QII-O22-RM2	—	—	—	—	—	—	—	—	—	—	—	—
QII-O22-LP3	18.0	18.5	0.7	0°	30°	28°	58°	58.0	109.0	170°	14.5	1.0
QII-O22-LM3	—	—	—	—	—	—	—	—	—	—	—	—
QII-O22-RP3	20.0	18.7	1.8	-5°	36°	15°	51°	73.0	117.0	155°	16.0	1.1
QII-O22-RM3	—	—	—	—	—	—	—	—	—	—	—	—
QII-O22-LP4	20.7	16.8	2.4	0°	—	—	—	54.0	—	146°	10.0	1.2
QII-O22-LM4	—	—	—	—	—	—	—	—	—	—	—	—
QII-O22-RP4	19.5	18.3	7.7	-10°	26°	32°	58°	—	103.0	—	—	1.1
QII-O22-RM4	5.3	4.5	0.6	—	—	—	—	—	—	—	—	1.2
QII-O22-LP5	—	—	—	—	—	—	—	—	—	—	—	—
QII-O22-LM5	—	—	—	—	—	—	—	—	—	—	—	—
QII-O22-RP5	24.5	18.7	1.9	-10°	—	—	—	67.0	—	—	—	1.3
QII-O22-RM5	5.5	6.7	1.0	—	—	—	—	—	—	—	—	0.8
QII-O22-LP6	—	—	1.5	—	—	—	—	—	—	—	—	—
Mean-P	20.6	18.9	2.4	|6°|	32°	30°	61°	62.8	109.4	160°	11.9	1.1
Mean-M	5.0	4.5	0.6	—	—	—	—	—	—	—	—	1.3
Remarks	RM2, LM3 and RM3 are missing; LM5 is possibly overprinted.											
QII-O23-LP1	19.3	19.8	1.6	-20°	38°	35°	73°	62.0	124.0	—	—	1.0
QII-O23-LM1	—	—	—	—	—	—	—	—	—	—	—	—
QII-O23-RP1	22.0	20.7	0.8	-20°	32°	33°	65°	63.0	121.0	170°	6.0	1.1
QII-O23-RM1	3.7	1.9	0.2	—	—	—	—	—	—	—	—	1.9
QII-O23-LP2	21.0	19.5	1.7	-25°	32°	34°	66°	59.0	—	170°	4.5	1.1
QII-O23-LM2	—	—	—	—	—	—	—	—	—	—	—	—
QII-O23-RP2	20.3	20.0	1.6	-15°	19°	50°	69°	—	—	—	—	1.0
Mean-P	20.7	20.0	1.4	-20°	30°	38°	68°	61.3	122.5	170°	5.3	1.1
Mean-M	3.7	1.9	0.2	—	—	—	—	—	—	—	—	1.9
Remarks	LM1 and LM2 are missing											
QII-O24-RP1	23.5	22.3	1.4	—	36°	32°	68°	63.5	131.3	155°	—	1.1
QII-O24-RM1	5.5	6.9	1.7	—	—	—	—	64.8	—	—	—	0.8
QII-O24-LP2	21.0	20.5	1.1	—	38°	29°	67°	70.6	136.5	161°	—	1.0
QII-O24-LM2	5.0	4.0	0.4	—	—	—	—	—	—	—	—	1.3
QII-O24-RP2	22.0	21.0	1.6	—	33°	28°	61°	67.6	—	—	—	1.0
QII-O24-RM2	(6.7)	(5.2)	0.5	—	—	—	—	—	—	—	—	—
QII-O24-LP3	25.0	23.0	0.6	—	—	—	—	—	—	—	—	1.1
Mean-P	22.9	21.7	1.2	—	35°	30°	64°	67.2	133.9	158°	—	1.1
Mean-M	5.3	5.5	0.9	—	—	—	—	64.8	—	—	—	1.1
Remarks	LM1 and LM3 are missing.											
OIII-O1-LP1	24.3	—	—	-19°	—	—	—	77.0	133.2	171°	—	—
OIII-O1-RP1	21.8	20.0	—	5°	30°	27°	57°	56.5	115.0	168°	—	1.1
OIII-O1-LP2	23.0	18.7	—	—	25°	23°	48°	61.6	—	171°	—	1.2
OIII-O1-RP2	24.2	18.1	—	—	24°	27°	51°	—	—	—	—	1.3
OIII-O1-LP3	—	—	—	—	—	—	—	—	—	—	—	—
Mean-P	23.3	18.9	—	|12°|	26°	26°	52°	65.0	124.1	170°	—	1.2
Remarks	Pes-only trackway.											
OIII-O2-RP1	22.2	17.0	—	5°	27°	29°	56°	50.8	98.7	180°	—	1.3
OIII-O2-LP2	21.7	18.5	—	2°	27°	26°	53°	48.5	100.0	167°	—	1.2
OIII-O2-RP2	20.0	17.8	—	7°	26°	30°	56°	52.8	104.5	175°	—	1.1
OIII-O2-LP3	22.0	17.7	—	-11°	29°	25°	54°	52.8	101.1	167°	—	1.2
OIII-O2-LM3	4.3	4.6	—	—	—	—	—	—	—	—	—	0.9
OIII-O2-RP3	21.8	19.4	—	4°	24°	26°	50°	49.4	99.8	167°	—	1.1
OIII-O2-LP4	21.3	17.0	—	0°	27°	27°	54°	51.3	100.5	173°	—	1.3
OIII-O2-RP4	20.8	18.2	—	—	38°	29°	57°	49.6	99.6	164°	—	1.1
OIII-O2-RM4	3.4	5.0	—	—	—	—	—	54.6	—	—	—	0.7
OIII-O2-LP5	21.5	17.0	—	—	22°	39°	51°	51.2	—	—	—	1.3
OIII-O2-LM5	3.2	5.1	—	—	—	—	—	—	—	—	—	0.6
OIII-O2-RP5	—	—	—	—	—	—	—	—	—	—	—	—
Mean-P	21.4	17.8	—	|5°|	28°	29°	54°	50.8	100.6	170°	—	1.2
Mean-M	3.6	4.9	—	—	—	—	—	54.6	—	—	—	0.7
Remarks	Only the LP3, RP4 and LP5 tracks with the manus traces.											
OIII-O3-RP1	18.2	15.1	—	-9°	23°	27°	50°	41.5	83.3	177°	—	1.2
OIII-O3-RM1	2.6	4.8	—	49°	—	—	—	45.7	84.8	157°	—	0.5
OIII-O3-LP2	17.5	14.8	—	—	24°	28°	52°	43.0	—	—	—	1.2
OIII-O3-LM2	3.2	4.3	—	—	—	—	—	41.3	—	—	—	0.7
OIII-O3-RP2	16.3	13.3	—	—	26°	30°	56°	—	—	—	—	1.2
OIII-O3-RM2	3.2	5.2	—	—	—	—	—	—	—	—	—	0.6
Mean-P	17.3	14.4	—	-9°	24°	28°	53°	42.3	83.3	177°	—	1.2
Mean-M	3.0	4.8	—	49°	—	—	—	43.5	84.8	157°	—	0.6
Remarks	Three well-preserved pes-manus pairs.											
OIII-O4-RP1	20.1	—	—	—	—	—	—	46.4	—	—	—	—
OIII-O4-LP2	17.0	14.5	—	—	19°	32°	51°	—	—	—	—	1.2
OIII-O4-LM2	3.0	5.0	—	—	—	—	—	—	—	—	—	0.6
Mean-P	18.6	14.5		—	19°	32°	51°	46.4	—	—	—	1.2
Mean-M	3.0	5.0	—	—	—	—	—	—	—	—	—	0.6
Remarks	RP1 lose the manus trace and digit II trace.											

Abbreviations: L: Maximum length; W: Maximum width; D: Maximum depth; II-III, III-IV, II-IV: angle between digits II and III, III and IV, II and IV; PL: Pace length; SL: Stride length; PA: Pace angulation; L/W: Dimensionless. GO: Remarks. In all spreadsheets positive pes and manus track rotation = outward rotation, negative pes and manus track rotation = inward rotation. () indicate that a given value has a high uncertainty. Generally, this is related to poor or incomplete track preservation.

#### Paratypes

Specimens, QII-O20-RP1–RM1, and LP2–LM2 comprise two manus-pes sets of natural mold tracks in the same trackway as the holotype (Fig 5, Table 1). Specimens, QII-O9-LP4–LM4, RP4–RM4, and LP5–LM5 comprise three manus-pes sets. As with the holotype, these specimens remain in the field. However, one manus-pes set is preserved as a rubber mold and replica in the University of Colorado collections as UCM 214.256.

#### Locality and horizon

Lotus tracksite, Qijiang, Chongqing, Lower Cretaceous (Barremian–Albian), Jiaguan Formation, China.

#### Emended diagnosis

Large size (~35 cm) quadrupedal ornithopod tracks. Pes trace mesaxonic, functionally tridactyl, with quadripartite morphology, consisting of impressions of three digits and a heel pad separated by pronounced ridges. Mean length /width ratio 1.1. Mean value of mesaxony, measured as L/W of anterior triangle 0.37. Manus trace suboval to semicircular, situated anterolaterally to pes trace, sometimes with faint traces of anteromedially positioned digit. Typical heteropody (ratio of manus to pes size) 1:6.1.

### Description

The Lotus tracksite reveals thirty-seven ornithopod trackways, catalogued as QI-O1‒O6, QII-O1‒O24 and QIII-O1‒O7; at least thirty-eight isolated ornithopod tracks were also preserved on the second layer. All these tracks are natural molds (concave epireliefs). Lotus ornithopod pes traces are ~15 cm to ~48 cm in length. Trackways QII-O1, O2, O4, O7, O9, O11, O13, O14 and O17‒O21 are longer than 30 cm and are referred to as Type A. Other pes traces shorter than 30 cm are referred to Type B. The authors take two sets of representative tracks from Type A and Type B for detailed description.

Type A (Figs 5 and 6; Tables 1 and 2): QII-O20 is the holotype trackway, revealing the most complete sequential manus-pes sets. The average L/W ratio is 1.2 for the pes and 0.7 for the manus. The manus impression is rotated approximately 46° outward from the trackway axis. This outward rotation is much larger than the inward rotation of the pes impressions (approximately 5°). The average manus PA is 149°, while the average pes PA is 165°.

**Table 2:** Measurements (in cm) of isolated ornithopod tracks from the Lotus tracksite, Chongqing Municipality, China.

Number.	L	W	II-IV	L/W
QII-OI1	33.5	33.0	57°	1.0
QII-OI2	36.0	32.5	—	1.1
QII-OI4	27.5	30.0	69°	0.9
QII-OI5	21.0	18.0	49°	1.2
QII-OI6p	27.0	23.8	59°	1.1
QII-OI6m	4.5	6.0	—	0.8
QII-OI7	11.0	21.0	—	0.5
QII-OI10	13.0	22.3	—	0.6
QII-OI11	26.0	23.0	65°	1.1
QII-OI12	19.5	13.5	61°	1.4
QII-OI14	32.5	30.0	62°	1.1
QII-OI15	20.0	19.4	—	1.0
QII-OI16	23.0	22.0	41°	1.0
QII-OI17	>11.0	—	—	—
QII-OI18	26.0	22.0	43°	1.2
QII-OI21	28.5	26.5	61°	1.1
QII-OI22	30.0	32.5	52°	0.9
QII-OI23	32.0	27.0	53°	1.2
QII-OI24	25.0	20.4	54°	1.2
QII-OI25	33.5	30.5	51°	1.1
QII-OI27m	7.0	9.0	—	0.8
QII-OI27p	30.5	21.0	43°	1.5
QII-OI29	17.0	17.5	68°	1.0
QII-OI30m	4.5	6.0	—	0.8
QII-OI30p	25.5	21.8	60°	1.2
QII-OI31m	9.5	11.0	—	0.9
QII-OI31p	31.0	29.0	54°	1.1
QII-OI33	32.5	27.5	48°	1.2
QII-OI34	20.0	22.5	—	0.9
QII-OI35	30.0	27.0	51°	1.1
QII-OI36	34.5	26.0	49°	1.3
QII-OI37	24.0	21.3	56°	1.1
QII-OI38	26.5	22.0	52°	1.2

The holotype pes-manus couple QII-O20-RP2-RM2 is the best-preserved example (Fig 6). The pes trace RP2 is mesaxonic, functionally tridactyl and plantigrade with a length of 30.5 cm, and shows quadripartite morphology, consisting of impressions of three digits and a heel pad separated by pronounced ridges, which, in life, represented well defined concave-up creases that separated the convex-down pads, as seen in natural casts which represent close approximations of foot replicas. The L/W ratio is 1.2 while the anterior triangle L/W ratio is 0.29. The digit III trace is the shortest, but most anteriorly-situated, while traces of digits II and IV are longer and almost equal in length. Each digit trace has a strong and blunt claw or ungual mark, which in the lateral digit is sharper than in digit III. The heel is sub-triangular. There is a distinct border between the heel and the three digits. The interdigital divarication II–IV is 57°. The divarication angle between digits II and III (28°) is almost equal to the one between digits III and IV (29°). The manus trace is oval, with no discernable digit or claw marks. The short axis of the oval manus trace aligns with the antero-lateral margins of the pes (i.e. aligned almost with the tip of digit III trace). The ratio of manus centre-pes center distance/pes trace length (mc-pcD/p’L) is 0.8. The heteropody (ratio of manus to pes size) is 1:6.1.

QII-O9 is a paratype trackway, andone of the best-preserved examples of Lotus ornithopod tracks. RP4-RM4 is the best preserved set. QII-O9 tracks are basically consistent with the holotype morphology, with an average L/W ratio of 1.1, and an average anterior triangle L/W ratio of 0.38 for the pes. A digit II claw trace can be seen in RM4. The mc-pcD/p’L ratio is 1.0. The heteropody is 1:5.6

In other large-sized sets, LM4, a manus track of QII-O14, has three depressions which may correspond to traces of digits II, III and IV. It is similar to the manus impression seen in a specimen from Lamar, Colorado (Denver Museum of Natural History #1608) [33]. RP2, a pes trace of QII-O14, has two associated manus tracks which might be formed by two consecutive steps when the trackmaker lost balance. Several tracks (e.g. O11-RP2, O21) show considerable morphological variation, which is probably due to the original substrate being wet and slippery.

**Fig 7:**
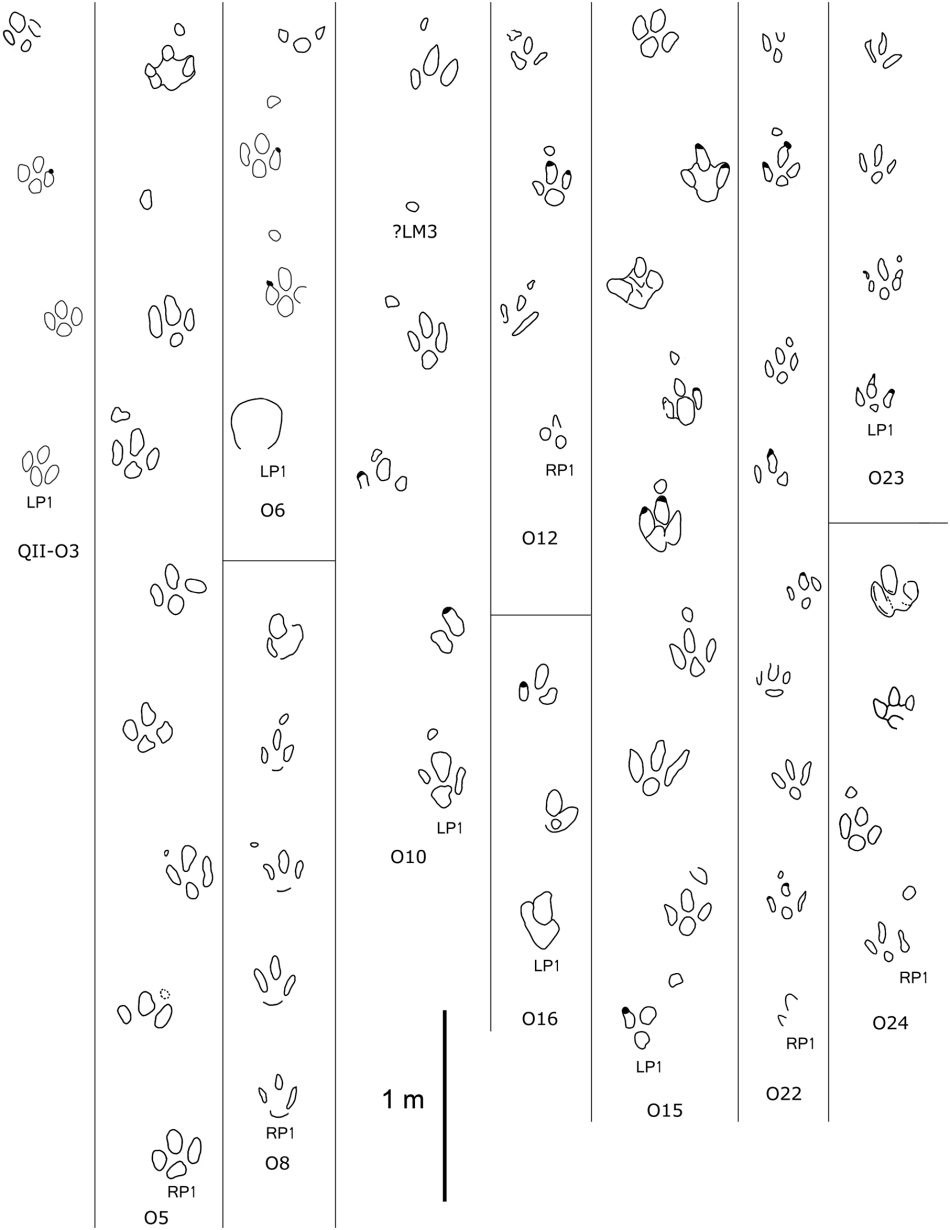
Interpretative outline drawings of small-sized ornithopod trackways from QII, Lotus tracksite, Qijiang, China.

**Fig 8:**
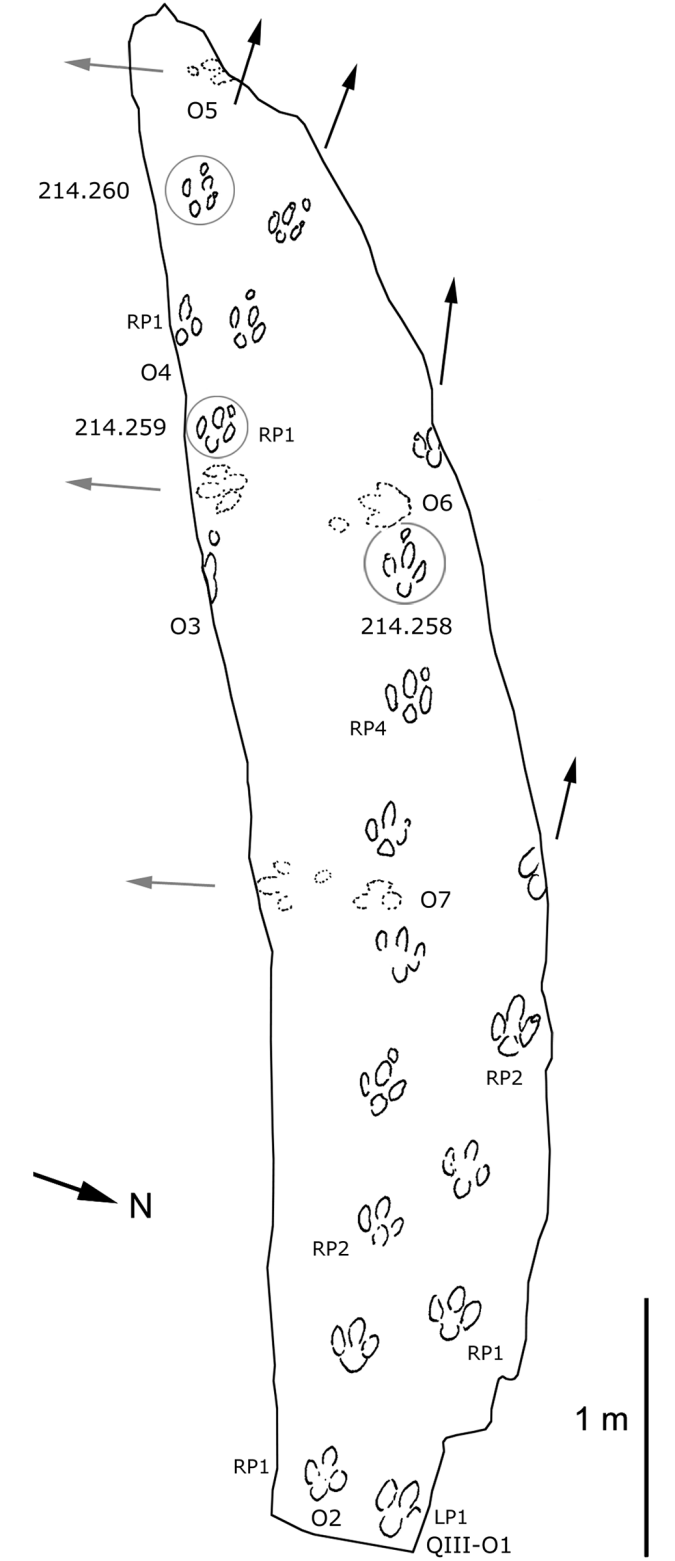
Interpretative map based on drawings of small-sized ornithopod trackways from QIII, Lotus tracksite, Qijiang, China. Note that specimens UCM 214.258–214.260 are preserved as replicas in the University of Colorado collections.

Type B (Figs 6–8; Tables 1 and 2): these small-sized tracks are only half as deep as Type A or less. Most of them are poorly preserved, however, some display excellent morphological features. QII-O3 and O6 are the best preserved and most representative. The pes-only QII-O3 trackway represents one of the smallest ornithopod individuals from the Lotus tracksite. The mean length of the QII-O3 trackway is 20.6 cm; the average ML/MW ratio is 1.2; the pes impression is rotated approximately 21° inward from the trackway axis. QII-O3-RP1is best preserved with an average ML/MW ratio of 0.9; digits II–IV are similar in length and digit II has the most developed ungual mark; the interdigital divarication II–IV is 63°; the divarication angle between digits II and III (32°) is almost equal to the one between digits III and IV (31°); the anterior triangle L/W ratio is 0.33.

The pes-manus association QII-O6-LP2–LM2 is also one of the best-preserved examples. Digit III is the shortest, digit III and IV are almost the same in length, and digit II has the most developed ungual mark. The anterior triangle L/W ratio is 0.27. The manus trace is almost semicircular in shape. The manus trace is situated anterior to pes digit III. The mc-pcD/p’L ratio is 1.3, the heteropody 1:8.3.

All seven trackways of QIII belong to type B, among which QIII-O1–O4 are true well-preserved tracks and O5–O7 are undertracks. The walking direction of the former trackmaker group was from east to west and that of the latter group from north to south. These tracks are morphologicallysimilar to Type B tracks (QII-O3, O6) from level II.

OI17 is the smallest pes. OI17 shows only one complete lateral digit and most part of digit III, while the remaining portion is overprinted by QII-O20-RP2. Based on complete type B specimens, OI17 is probably 11–12 cm in length, less than a third the size of type A, and is likely a juvenile.

### 2 Remarks

**Fig 9:**
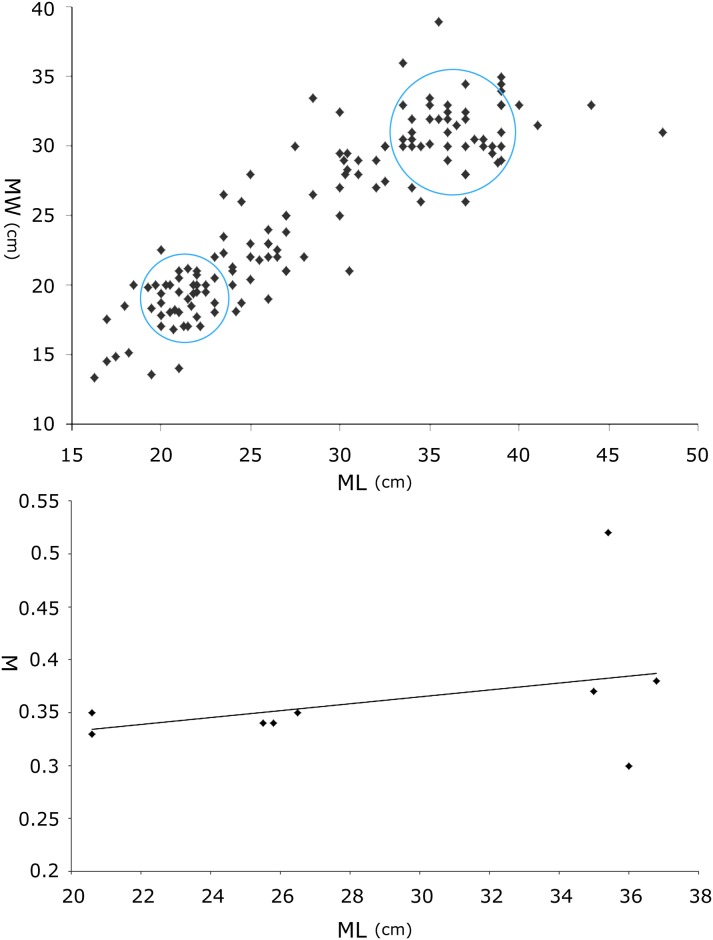
Scatter diagram of track length and width (A); length and mesaxony (B) in *Caririchnium* tracks from the Lotus tracksite.

1. In well-defined ichnotaxa, sizes of tracks can reflect the size and age of the individual trackmakers [34–35]. The strong similarity in morphology suggests that type B tracks probably represent juveniles or subadults of type A. The scatter diagram (Fig 9, Table 3) of length and width of the pes tracks shows that most tracks fall in the ranges of 20–24 cm and 33–37 cm, and this likely reflects two age cohorts, although other explanations are possible (e.g., sexual dimorphism).
2. Generally, the manus impressions are strongly rotated outward from the trackway axis, and the pes impressions are rotated slightly inward.
3. The L/W and PL/L of all pes tracks are similar, with consistent averages (1.1) and medians (2.6).
4. In type B, only QII-O3, O16, and OIII-O1 lack manus traces, if not due to bipedal gait, possibly because the original manus tracks were too shallow to be preserved. Xing et al. [2] considered the possibility that subadult trackmakers usually walked only on their hind feet [36–37], but there is no unequivocal ichnological or osteological evidence to support this interpretation.
5. In type A tracks, the axis of the manus trace always aligns with the antero-lateral margins of the pes trace. However, in type B tracks, the positions of the manus traces appear more variable. In addition to those with similar configurations to type A, others align more with the anterior margins of the pes trace: i.e., anterior to the distal end of digit III.
6. Mesaxony (0.33–0.35) of pes tracks in type B is slightly smaller than that (0.37–0.52) of type A (Fig 9, Table 4). Thus, there is a slight tendency towards elongation of the anterior triangle (stronger mesaxony) in the smaller tracks in group B. This is consistent with observations by Lockley [38] and references therein.
7. The area ratio of pes and manus tracks in type B is generally smaller than type A, so adult trackmakers had higher heteropody.
8. Most Lotus ornithopod trackmakers from levels I and II went from west to east, suggesting that this area was a possible thoroughfare for trackmakers perhaps controlled by some physical landscape features [39]. The west-to-east direction may have been parallel to a river course or shoreline [40]. The bird and pterosaur tracks have the same general orientation. Bird trackways run parallel to the interpreted direction of the river’s flow [4]. This suggests that the trackmakers may have been foraging, as has been observed in *Goseongornipes* isp. [41] and *Koreanaornis* cf. *hamanensis* trackways [42].

**Table 3:** Summary of size data for *Caririchnium lotus* from theLotus tracksite, Qijiang, Chongqing Municipality, China. (n = 140).

Measurement	Range	Mean	Median	StandardDeviation
**L (cm)**	16.3–48.0	28.6	27.0	7.2
**W (cm)**	13.3–39.0	25.1	25.0	6.0
**L/W**	0.9–1.5	1.1	1.1	0.1
**PL/L**	2.0–4.2	2.6	2.6	0.4

**Table 4:** Summary of size (cm) and mesaxony data for *Caririchnium lotus* from the Lotus tracksite, Qijiang, Chongqing Municipality, China.

No.	L	M
QII-O9	36.8	0.38
QII-O11	35.0	0.37
QII-O14	35.4	0.52
QII-O20	36.0	0.30
QII-O3	20.6	0.35
QII-O5	26.5	0.35
QII-O6	25.5	0.34
QII-O15	25.8	0.34
QII-O22	20.6	0.33

### 3 Comparisons and discussion

Lower Cretaceous ornithopod trackways are well-known from Europe, North America, and East Asia. To date, six valid ornithopod ichnogenera have been named from the Cretaceous: *Amblydactylus* (two ichnospecies), *Caririchnium* (four ichnospecies), *Iguanodontipus*, and *Ornithopodichnus* from the Lower Cretaceous, and *Hadrosauropodus* (two ichnospecies) and *Jiayinosauropus* [31] from the Upper Cretaceous. For historical reasons, three ichnogenera first named *Amblydactylus*, *Caririchnium* and *Iguanodontipus* were inferred to have been made by ornithopods of the genus *Iguanodon*, or similar iguanodontian trackmakers. Lockley et al. [31] referred them to the ichnofamily Iguanodontipodidae based on morphological similarity.

**Fig 10:**
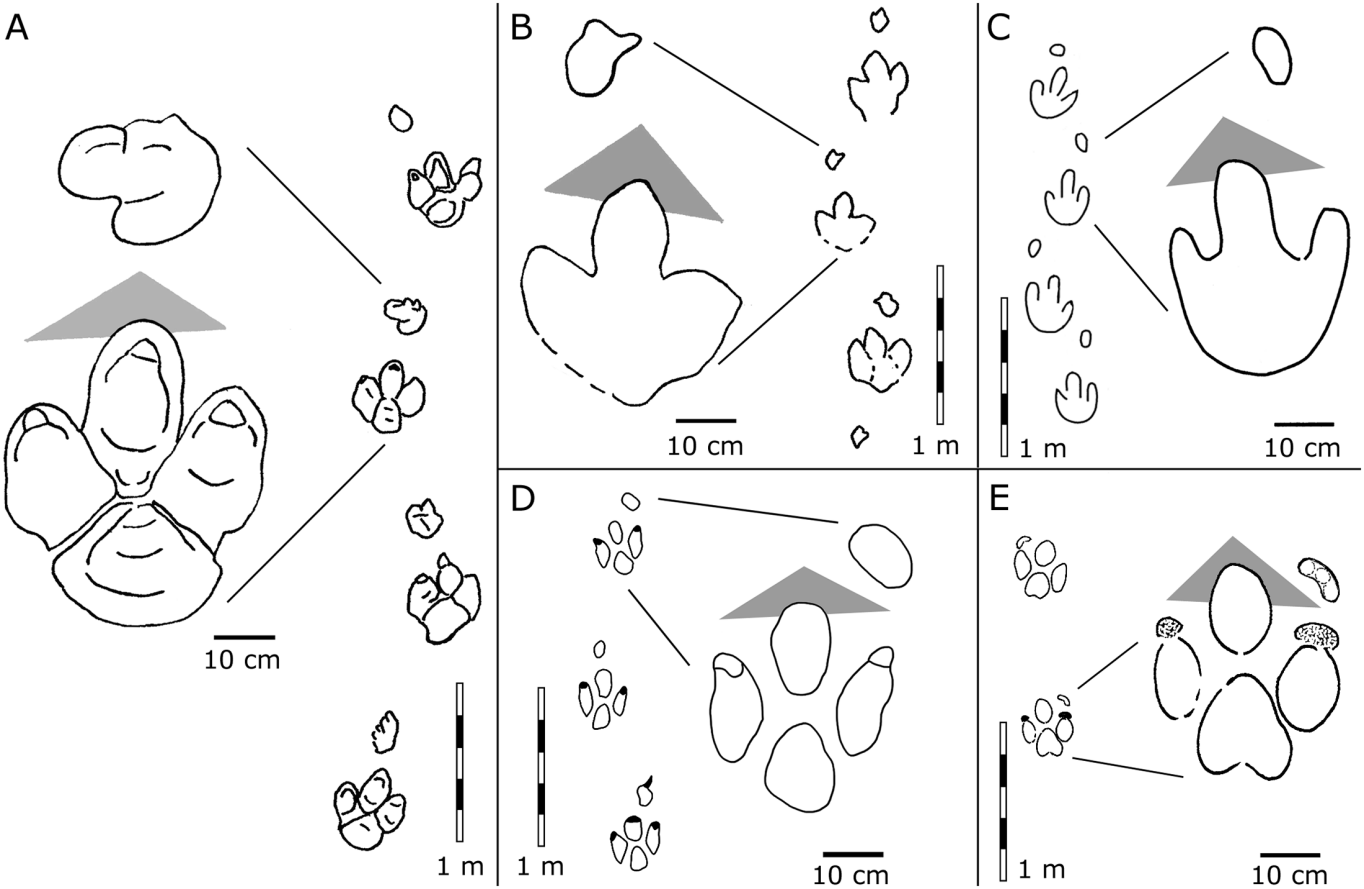
Interpretative outline drawings of *Caririchnium* drawn to the same scale (modified after Lockley et al.[31]). A, *Carirchnium magnificum* [32]; B, *Caririchnium leonardii* [33]; C, *Caririchnium protohadrosaurichnos* [46]; D, *Caririchnium lotus* [2], and E, *Caririchnium kyoungsookimi* [47].

The holotype trackway of *Caririchnium* was originally named by Leonardi [32]. *C*. *magnificum* is based on a well-preserved trackway of a quadruped from the Antenor Navarro Formation (Fig 10), in the lower part of the Rio do Peixe Group (pre Aptian) of Brazil [43]. The mesaxony of the type specimen of *C*. *magnificum* is 0.31; the heteropody is 1:3.7. The former value is lower than that measured for the Lotus specimens, whereas the latter is higher. The manus traces of *C*. *magnificum* are irregular in size and shape, ranging from a large and irregular ‘L’ shaped trace to oval or subcircular.

The second ichnospecies of *Caririchnium*, *C*. *leonardii* from the upper part of the Dakota Group (Albian–Cenomanian) of Colorado, USA, [31, 33, 44], is similar to *C*. *magnificum* in overall morphology, however, *C*. *leonardii* differs from *C*. *magnificum* in the configuration of the manus and the shape of the heel [31]. Some specimens later referred to *C*. *leonardii* are well preserved, with skin impressions, such as trackway MWC 201.1 from the South Platte Formation (Late Aptian-Early Cenomanian) of the Dakota Group of Colorado, USA [45]. MWC 201.1 has a bilobed (2-lobed) heel. Moreover, the mesaxony of the type specimen of *C*. *leonardii* is 0.46 and the heteropody is 1:8.1. As such, *C*. *leonardii* has stronger mesaxony and weaker heteropody than the Lotus specimens.

The holotype of the ichnospecies *Caririchnium protohadrosaurichnos* [46] comes from the Woodbine Formation (Cenomanian) of Texas, USA. *C*. *protohadrosaurichnos* has a less defined quadripartite pes and a more elongate manus [31]. The mesaxony of the type specimen of *C*. *protohadrosaurichnos* is 0.39 (based on the holotype SMU 74653), and the heteropody is 1:14.6. The latter suggests an extremely small manus for *C*. *protohadrosaurichnos*. All these features differ to some degree from to those in the Lotus specimens.

*Caririchnium kyoungsookimi* is a quadrupedal ornithopod trackway from the Jindong Formation (Late Aptian) of Korea [47]. The manus shows three unusual subcircular indentations arranged in an elongate arc, a pattern which is unlike any seen in other *Caririchnium* ichnospecies, although it is somewhat similar to ornithopod tracks from the basal Cretaceous of Germany [48].

Additionally, *Caririchnium* can be found in the Antlers Formation (Aptian-Albian) of Oklahoma [49], the Mesa Rica Sandstone and Pajarito formations (late Albian) of New Mexico [50–51] and the Patuxent Formation (Aptian) of Virginia[52] from USA;, the Jindong Formation (Late Aptian) of Korea [53] and the Amagodani Formation (Barremian) of Japan [54]. Lockley et al. [31] noted that, all holotype *Caririchnium* trackways preserve both manus and pes tracks, unlike other ornithopod holotype ichnotaxa, which are often based on single pes tracks. Thus, the *Caririchium* trackmakers were typically quadrupedal. The approximate age range of *Caririchnium* (similar to *Amblydactylus*) is ~Barremian–Cenomanian, which corresponds to the age of the Lotus specimens (Barremian–Albian).

### 4 *Caririchnium* from China

**Fig 11:**
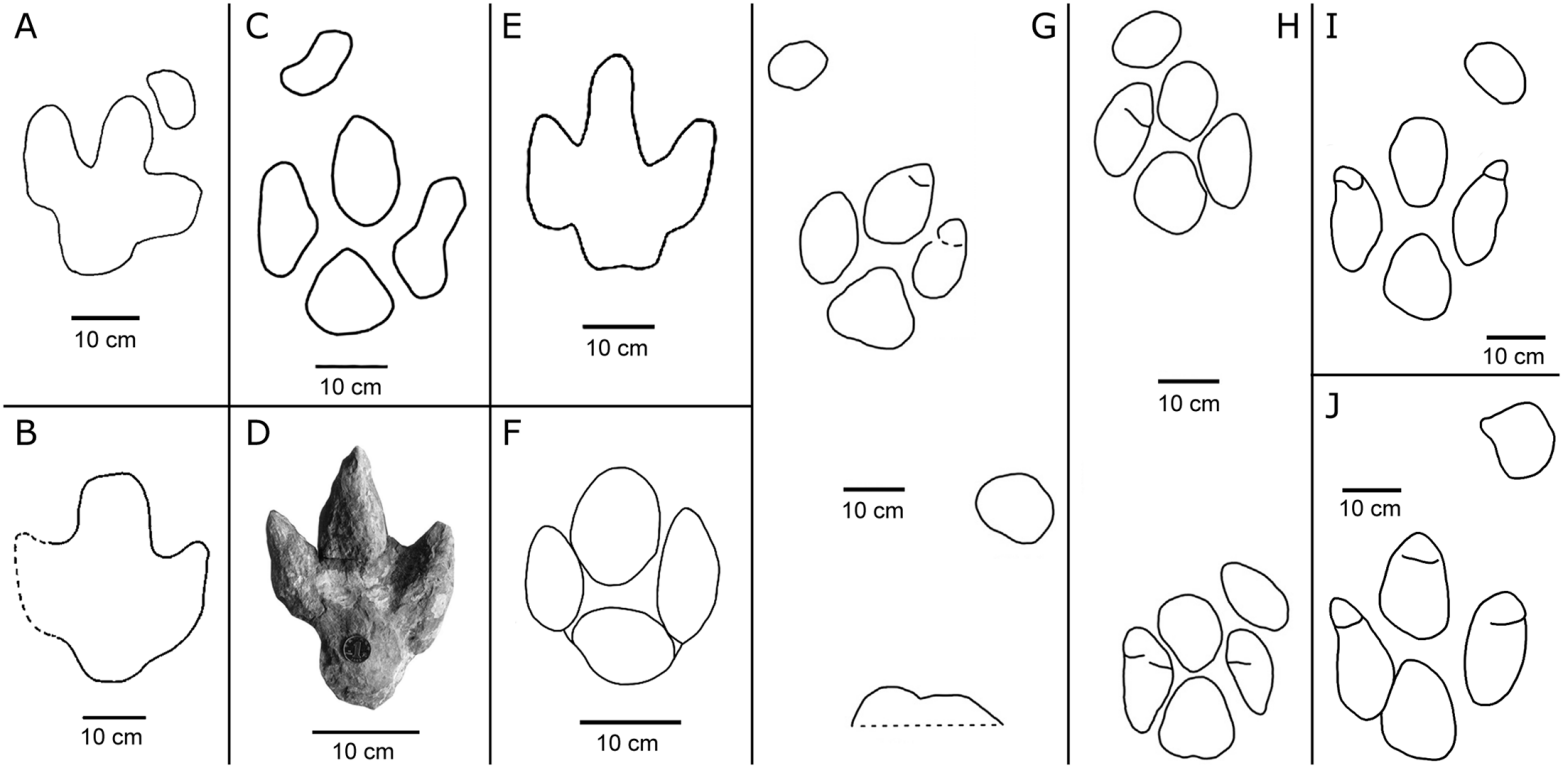
Interpretative outline drawings of Early Cretaceous *Caririchnium* tracks from China. A, *Caririchnium* isp. from Jiufotang Formation, Luanping tracksite, Hebei Province [55–56]; B, *Caririchnium* type from Tongfosi Formation, Tongfosi tracksite, Jilin Province [57]; C, *Caririchnium* from Hekou group, Yanguoxia tracksite No. II, Gansu Province; D, *Caririchnium* type from Hekou group, Yanguoxia tracksite No. I [58]; E, *Caririchnium* from Hekou group, SS1 site, Gansu Province [59]; F, *Caririchnium* from Jiaguan Formation, Longjing tracksite, Sichuan Province [76]; G and H, *Caririchnium* from Feitianshan Formation, Zhaojue tracksites, Sichuan Province [8]; I and J, this text. Scale bar = 10 cm.

Besides the Lotus tracksite, *Caririchnium* has already been found at five other tracksites of China (Fig 11).

1. *Caririchnium* isp. tracks from the Early Cretaceous Jiufotang Formation, Luanping tracksite, Hebei Province. You and Azuma [55] first reported these ornithopod trackways in a large exposure along a railway. Matsukawa et al. [56] mapped this tracksite and referred the tracks to *Caririchnium*. These tracks represent quadrupedal ornithopods and are mostly not very well preserved. The pes tracks show quadripartite morphology, and the mesaxony value is 0.29. The manus traces are oval, the short axis aligns with the antero-lateral margins of the pes trace. Slender-toed theropod tracks (*Asinodopodus*) are also found at this tracksite.
2. *Caririchnium* type tracks from the Early Cretaceous or early Late Cretaceous Tongfosi Formation, Tongfosi tracksite, Yanji City, Jilin Province [57]. These tracks are poorly preserved. Manus track are absent and the pes track shows possible quadripartite morphology. Gracile theropod tracks are also found at this tracksite.
3. *Caririchnium* type tracks from the Early Cretaceous Hekou group, Yanguoxia tracksites, Gansu Province [58–59]. At the Yanguoxia tracksites, ornithopod tracks are preserved as in situ trackways and as natural casts. The trackways from site II and site VI are typical *Caririchnium*. The mesaxony value is 0.30. These tracks show that the trackmakers were gregarious. The inter-trackway spacing is fairly regular at about 1.3 m. Thus, only about 4 m separates the four trackways [58]. These tracks represent bipedal and quadrupedal ornithopods.
  GDM-Y-SS1-1 from Yanguoxia site SS1 and a natural cast from site I [58] have relatively strong mesaxony values, reaching 0.38 and 0.41, respectively. However, the natural casts are similar to *Caririchnium* in overall morphology, including quadripartite morphology and oval or triangular heel pad with a bilobed posterior margin. Such difference may arise from variable individual development or preservation factors and requires further study.
4. *Caririchnium* from the Early Cretaceous Jiaguan Formation, Longjing tracksite, Sichuan Province [60]. All trackways from Longjing tracksite occur on a sandstone bedding plane in a river bed, and, consequently, are subject to continued erosion. The ornithopod trackway that lacks a manus imprint is assigned to *Caririchnium* [60]. The best preserved LJ-O1-R1 is 22 cm long and has a mesaxony value of 0.34. Generally, it is quite similar to *Caririchnium lotus* type B. Digit II is the shortest but thickest, but this feature may be the result of weathering.
5. *Caririchnium* from the Lower Cretaceous Feitianshan Formation, Zhaojue tracksites, Sichuan Province [8]. The Zhaojue *Caririchnium* pes tracks have lengths ranging between about 20–30 cm, and indicate quadrupeds or facultative bipeds, among which the best preserved ZJII-O98 and O99 are generally similar to *Caririchnium lotus* with mesaxony values of 0.34–0.38. The *Caririchnium* trackmakers were almost certainly gregarious. Besides *Caririchnium*, *Ornithopodichnus* corresponding to smaller bipeds are also found at the Zhaojue tracksites.

So, the large ornithopod tracks from the Lotus, Longjing and Zhaojue sites of the Lower Cretaceous Sichuan-Yunan Basin are morphologically similar to, and most likely belong to, *Caririchnium lotus*. Numerous tracksites demonstrate that ornithopods were flourishing in the basin during the Early Cretaceous. Contemporary large ornithopod tracks from Lanzhou-Minhe Basin and Northeast China require further comparison.

In this regard it is worth noting the extensive samples of *Caririchnium* reported from the Cretaceous of Korea [61]. In many cases the abundant Korean assemblages show up to several dozen parallel trackways, with regular inter-trackway spacing, which strongly indicate gregarious behavior. Most of the Korean trackways indicate bipedal progression.

### 5 Speed estimates

Thulborn [62] suggests that for large ornithopods (p’L > 25 cm) h = 5.9* p’L and that for small ornithopods (p’L < 25 cm) h = 4.8* p’L. The relative stride length (SL/h) may be used to determine whether the animal is walking (SL/h≤ 2.0), trotting (2<SL/h<2.9), or running (SL/h≥2.9) [62–63]. The SL/h ratios calculated for the Lotus ornithopod trackways type A range from 0.41 to 0.98 and accordingly suggest a walking gait. Using the formula of Alexander [63], the speed of the trackmakers ranges between an estimated 1.12‒4.14 km/s. The type B trackways also indicates a walking speed, the SL/h ratios ranges from 0.98 to 1.72, and 2.59 and 6.91 km/s (Table 5). Obviously, minor Type B walked much faster than adult type A.

**Table 5:** Estimated data of the speed of Lotus ornithopod trackmakers.

large ornithopods (L > 0.25m)			small ornithopods (L < 0.25m)		
No.	SL/h	S (km/h)	No.	SL/h	S (km/h)
QII-O1	0.80	2.95	QII-O3	1.72	6.91
QII-O2	0.89	3.13	QII-O8	1.01	3.10
QII-O4	0.41	1.12	QII-O12	1.24	4.03
QII-O5	0.91	3.02	QII-O16	1.06	3.17
QII-O6	0.92	2.99	QII-O22	1.11	3.31
QII-O7	0.98	4.14	QII-O23	1.23	4.00
QII-O9	0.75	2.56	QII-O24	1.22	4.10
QII-O10	0.85	2.74	OIII-O1	1.11	3.56
QII-O11	0.74	2.48	OIII-O2	0.98	2.77
QII-O13	0.75	2.66	OIII-O3	1.00	2.59
QII-O14	0.82	2.95			
QII-O15	0.84	2.59			
QII-O17	0.66	1.98			
QII-O18	0.87	3.38			
QII-O19	0.91	3.38			
QII-O20	0.87	3.28			
QII-O21	0.88	3.35			

Abbreviations: SL/h, relative stride length; S = absolute speed

### 6 Preservation

*Caririchnium lotus* tracks from the Lotus tracksite are preserved in different ways, including typical impressions or molds (concave epireliefs), natural casts, (convex hyporeliefs) deep casts and undertracks. This variable preservation can help to give insight into morphological difference and variation between ornithopod tracks formed under different substrate conditions. However, most of the trackways of surface QII show exceptionally good preservation.

#### 6. 1 Toe-only tracks

**Fig 12:**
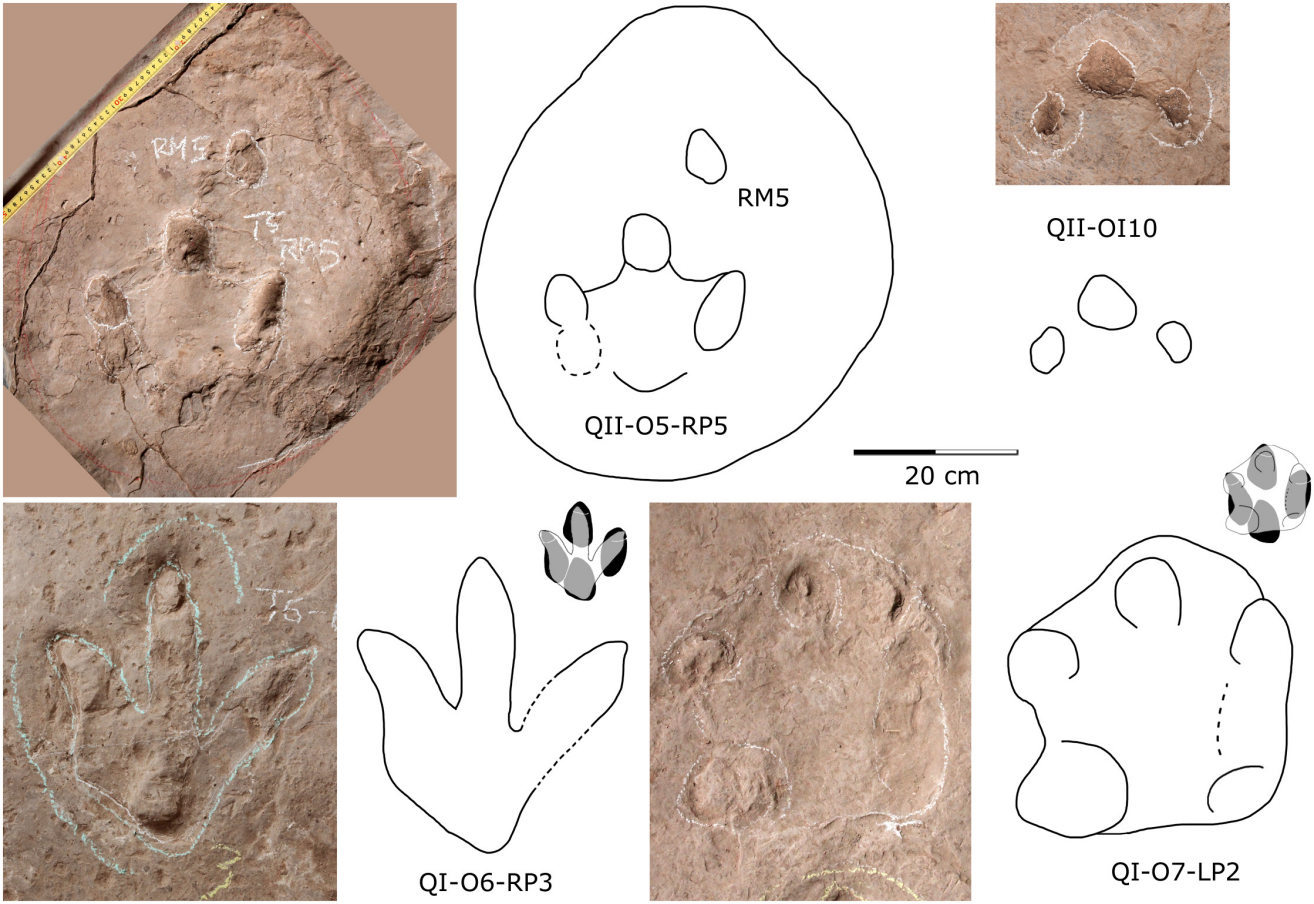
Photographs and interpretative outline drawings of ornithopod tracks and undertracks from the Lotus tracksite. These extramorphological variants of *C*. *lotus* formed the basis for two ichnospecies, which we reject here as *nomina dubia*. See text for details.

The second layer preserves about five isolated toe-only *Caririchnium* natural pes molds, with QII-OI10 being the most distinct (Fig 12). OI10 has three separated, rounded distal digit impressions but lacks a heel impression. Analyzing the well preserved *Caririchnium* trackway QII-O5 helps us to understand such toe-only tracks. QII-O5 RP1-RP4 show a well preserved quadripartite morphology consisting of impressions of three digits and a heel pad separated by pronounced ridges, while RP5 is overlapped by a sauropod pes undertrack which compressed the RP5 bearing sediment and lead to external morphological distortions of RP5. In this way, the relatively shallow part RP5 is flattened, and the tracks were turned from quadripartite tracks to toe-only pes prints. However, OI10 is not covered by a sauropod undertrack and was likely left when the substrate was relatively firm. Therefore, only the distal ends of the digits, which were generally more deeply impressed than the rest of the print, were registered. Alternatively, it could be the undertrack of an overlying track which disappeared due to denudation of sediment.

Tracks similar to OI10 were also discovered at the Early Cretaceous Yanguoxia tracksite in Yongjing, Gansu [64] and the Early Cretaceous Huangyanquan tracksite in Xijiang, China [65]. The Yanguoxia specimens are thought to have been made by an ornithopod, trackmaker on a partially submerged substrate, that propelled itself by toe-only steps, leaving a subaqueously registered trail [64, 66]. However, this interpretation is unduly complex, and we prefer to consider the toe-only traces as evidence of animals walking on the overlying bed (QII) while parts of their feet (toes) penetrated to the underlying layer (QI). The Huangyanquan specimens are interpreted as thyreophoran undertracks (possibly *Deltapodus curriei*) [65]. The discovery of Lotus toe-only *Caririchnium* natural pes prints may help to understand the origin (behavior or preservation) of such morphology.

#### 6.2 Misleading undertracks

*Caririchnium* natural molds are well-preserved on the second layer, due to suitable, even near optimal sediment and the presence of a microbial mat. *Caririchnium*, from the second layer, left undertracks on the first layer 10 cm below. These undertracks register different “morphological” (i.e. extramorphological) characteristics (Fig 12). OI-O6-RP3 has narrower digits II–IV, which are similar to theropod tracks. The heel of OI-O7-LP2 is quite shallow, while lateral digits (especially digit IV) have deep distal and proximal ends, forming a pentadactyl outline with digit III. This undertrack slightly resembles typical ankylosaur track morphology. Xing et al. [2] referred OI-O6-RP3 to ornithopod tracks naming it *Laoyingshanpus torridus*, and referred OI-O7-LP2 to ankylosaur tracks naming it *Qijiangpus sinensis*. Based on the present detailed study we conclude that such designations are not ichnotaxonomically useful or valid, and that these two ichnotaxa are *nomina dubia* [31], better interpreted as ornithopod undertracks, presumably transmitted to layer QI by the registration of *C*. *lotus* tracks on the overlying QII surface.

#### 6.3 Complexly overprinted series

**Fig 13:**
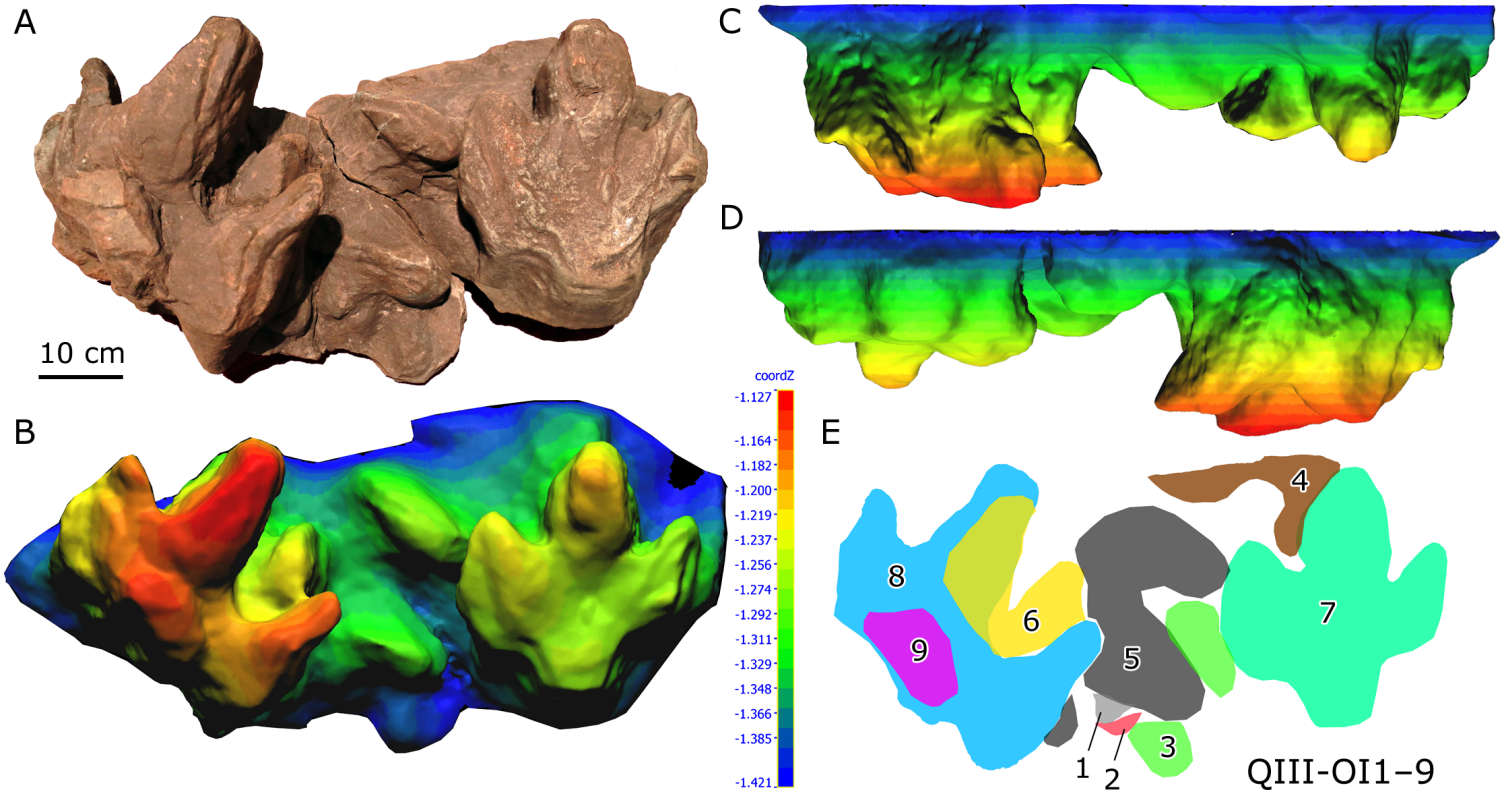
Photograph (A), 3D height maps (B–D) and interpretative outline drawing (E) of complexly overprinted ornithopod track series QIII-OI1–9 from the Lotus tracksite.

**Fig 14:**
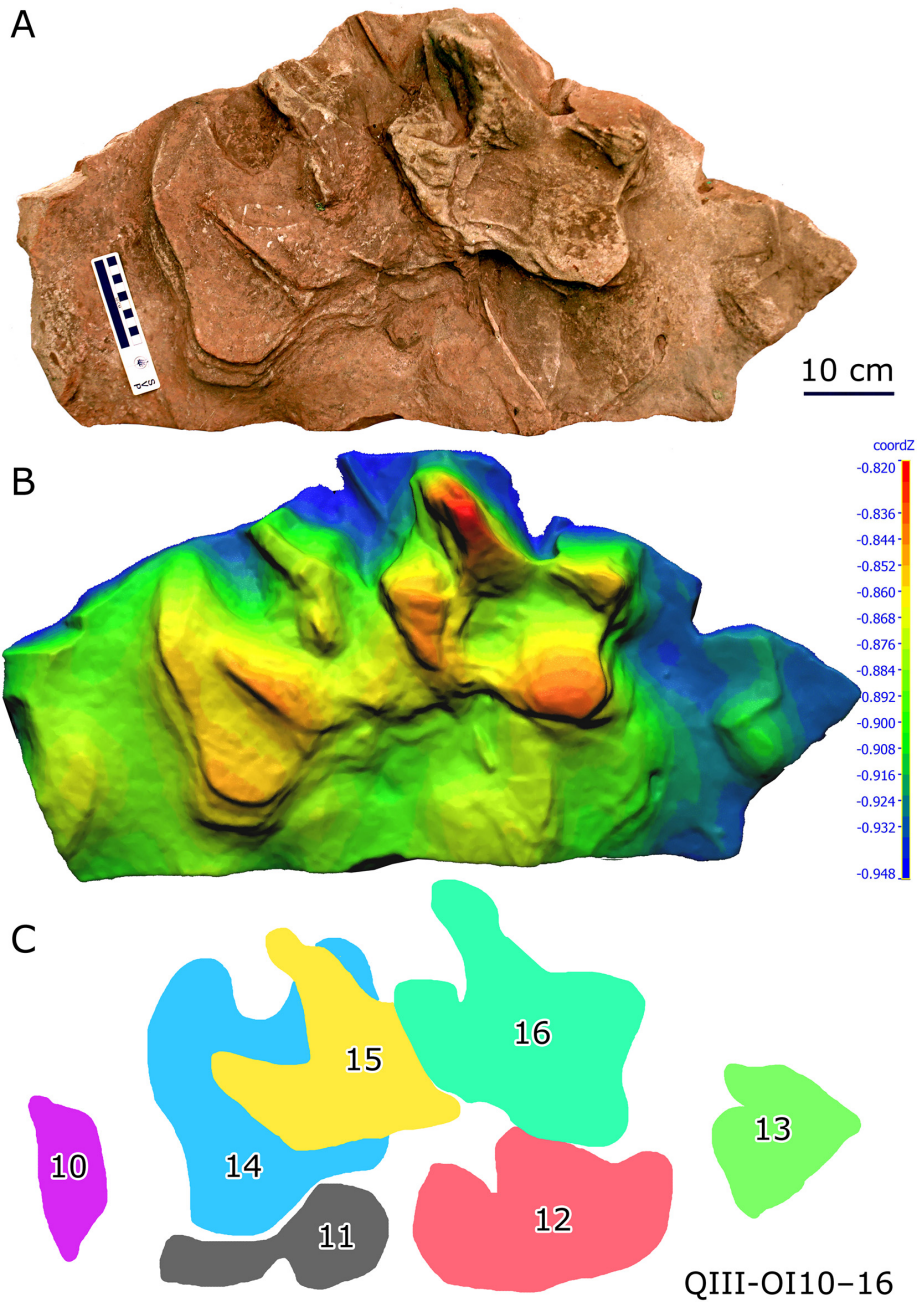
Photograph (A), 3D height map (B) and interpretative outline drawing (C) of complexly overprinted ornithopod track series QIII-OI10–16 from the Lotus tracksite.

Xing et al. [5] described the deep (three-dimensional) large ornithopod sandstone cast QIII-OI20 (former specimen number: QJGM-C1) from the third layer of the Lotus tracksite. This specimen provides unique insights into the locomotor mechanics of the trackmakers and permits reconstruction of the footfall, weight-bearing, and kick-off phases of the step cycle. The third layer also preserves two complex overprinted series, including nine and seven tracks, respectively (QIII-OI1-9 and QIII-OI10-16) (Figs 13 and 14). These two track series were made by multiple individuals travelling in different directions.

The 3D color topographic profiles help to sort out the sequences of QIII-OI1–9 and QIII-OI10–16. For example, OI1 and 2 were likely made first. Then OI3 and 4 were made and the former covered OI1 and 2. Then, OI5, 6, 8 and 9 were probably made, which overlapped and resulted in external morphological distortion of the earlier tracks. OI7 destroyed the edges of OI3 and 4. OI7 and OI8 are the best preserved casts. The former shows the principal *Caririchnium lotus* morphology, while the latter displays increased space between the lateral digit (right one) and digit III, probably attributable to the digits splaying outward in slippery sediment [5]. In addition, both OI7 and OI8 show digit III impressed considerably deeper than digits II and IV, a phenomenon that has been observed in some other hadrosaurid tracks [67].

QIII-OI10–16 and the especially shallow OI10–13 were likely made first, followed by overlapping OI14–16. Interestingly, the sediment bearing these tracks may have been compressed such that the tracks are flattened, especially OI14 and 15. During compaction, two layers of concentric outlines were formed on OI14. The flattened heel pad of OI16 is separated from the digital pad impression region by a shallower area.

Such interesting extramorphological variation shows that even tracks of the same ichnospecies can differ widely in shape due to different substrate conditions and various diagenetic processes.

## Sauropod Tracks

### 1 Undertracks

**Fig 15:**
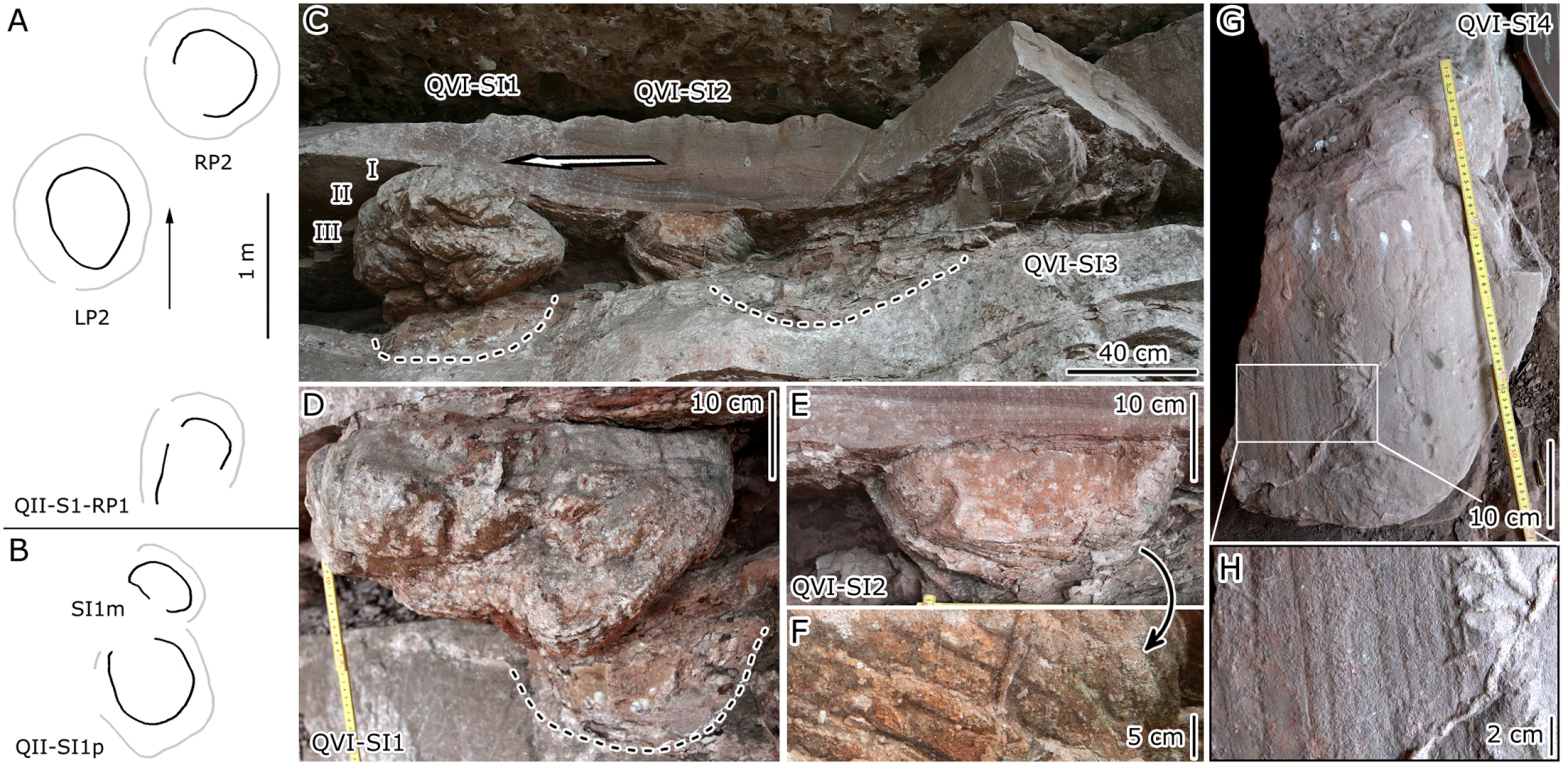
Interpretative outline drawings of sauropod trackway (A) and isolated pes-manus prints (B) from QI. Photograph (C, D, E and G) of sauropod casts from QVI. Close-up photographs (F, H) show details with striation marks. Dotted line indicates outline of undertracks. Arrow indicates moving direction of tracks.

Sauropod tracks from the Lotus tracksite have two preservation patterns: undertracks on the second layer and deep casts on the third and fifth–seventh layers (Fig 15, Table 6). The sediment of the third layer may have been soft and therefore most tracks are deep, such as the 37.1 cm deep *Caririchnium lotus* cast [5]. The feet of the trackmakers from the third layer caused strong distortion [68], leaving undertracks on the second layer. There are at least 20 broad, shallow depressions interpreted as undertracks transmitted onto the second layer. Three of these depressions form a pes only trackway catalogued as QII-S1, and four depressions form clear pes and manus traces, catalogued as QII-SI1p-1m–3p-3m.

**Table 6:** Measurements (in cm) of the sauropod tracks from Lotus tracksite, Chongqing Municipality, China.

Number.	L	W	R	PL	SL	PA	L/W	WAP	WAP/P’L
QII-S1-RP1	—	—	22°	183.0	256.0	116°	—	86.2	—
QII-S1-LP2	66.5	52.0	—	126.0	—	—	1.3	—	—
QII-S1-RP2	54.0	56.0	—	—	—	—	1.0	—	—
Mean	60.3	54.0	22°	154.5	256.0	116°	1.2	86.2	1.4
QII-SI1m	27.0	46.5	—	—	—	—	0.6	—	—
QII-SI1p	65.0	57.0	—	—	—	—	1.1	—	—

Abbreviations: L: Maximum length; W: Maximum; R: Rotation; PL: Pace length; SL: Stride length; PA: Pace angulation; WAP: Width of the angulation pattern of the pes (calculated value); L/W, and WAP/P'L are dimensionless.

The QII-S1 pes impression is oval with an average length of 60.3 cm and a L/W ratio of 1.2. The track turns outward by about 22°. The digit traces of QII-S1 are too indistinct to recognize with confidence, and the metatarso-phalangeal region is smoothly curved. QII-SI1p is oval and 65.0 cm in length, similar to QII-S1, and has a L/W ratio of 1.1. QII-SI1m is slightly U-shaped and has a L/W ratio of 0.6. Though these are only undertracks, they show the typical morphology of sauropod pes-manus prints [69–70].

For the trackways with both manus and pes traces, gauge (trackway width) was quantified for pes and manus tracks by using the ratio between the width of the angulation pattern of the pes (WAP) and the pes length (L) [71–72]. If the ratio is smaller than 1.0, tracks intersect the trackway midline, which corresponds to the definition of narrow-gauge [73]. Accordingly, a value of 1.0 separates narrow-gauge from medium-gauge trackways, whereas the value 1.2 has been used to distinguish between medium-gauge and wide-gauge trackways. The WAP/ML ratio of QII-S1 is 1.4, which is between medium-gauge and wide-gauge trackway. On the other hand, the factors affecting gauge may include the speed of the trackmaker [74–75] and the quality of preservation. In reference to this latter factor, it is important, as noted in the following section, to differentiate between true tracks with steep walls and well-defined outlines and undertracks (transmitted tracks are usually wider) with very low angle margins, which may reduce the inner trackway width and thus estimations of gauge [76]. If based on the displacement rim of QII-S1, the WAP/ML ratio is 1.1, more close to narrow-gauge.

Most sauropod trackways in China are wide- (or medium-) gauge and are therefore referred to the ichnogenus *Brontopodus* [70]. The wide-gauge of *Brontopodus*-type trackways suggests that the tracks were left by titanosauriform sauropods [70, 77]. The Lotus sauropod tracks are also consistent with the characteristics of *Brontopodus* type tracks from the Lower Cretaceous of the USA [69, 78]. These characteristics include medium-gauge/wide-gauge trackways, outwardly-directed pes tracks that are longer than wide, and a high degree of heteropody (ratio of manus to pes size). The heteropody ratio of 1:2.6 in QII-SI1 is close to 1:3 in *Brontopodus birdi*. Unfortunately, effective statistical evaluation is difficult due to the low relief and indistinct margins of the Lotus sauropod undertracks. Therefore, despite being measured as narrow-gauge, these tracks are provisionally assigned to the *Brontopodus-*type.

Theropod (with bird) and ornithopod tracks from the Jiaguan Formation were the only track types first described by Zhen et al. [1] from Emei Region. Sauropod tracks were not reported from that assemblage, a conclusion confirmed by Matsukawa et al. [56]. Although a local geologic report [79] mentioned a quadruped trackway, the tracks were not re-located in a 1982 field survey conducted by the Chongqing Natural Museum [80], or during the 2001 field investigation leading to the report of Matsukawa et al. [56]. Subsequently, narrow- and medium-gauge sauropod tracks were found at Hanxi [81] and Xinyang [76] tracksites. Hanxi specimens have WAP/ML ratios of 0.9 to 1.1, and the ratio of Xinyang specimens is 1.4. Therefore, sauropod tracks from the Jiaguan Formation are mostly narrow- and medium-gauge, consistent with Early Cretaceous sauropod tracks from other sites in south-central China, such as the Zhaojue tracksites in Sichuan Province [10], but differ from wide-gauge tracks of the Yanguoxia tracksite in Gansu Province [58]. This may imply that titanosauriform sauropods from different Early Cretaceous basins were distinct.

### 2 Natural casts

The sauropod track casts are deep natural tracks left in soft and moist substrates with a relatively high cohesiveness. They offer a glimpse into the three-dimensional foot morphology of the sauropod trackmakers and their foot movement (locomotion). Among the deep sauropod casts on the third and fifth–seventh layers, casts on the sixth layer are the best preserved and include at least four sauropod casts, catalogued as QVI-SI1–4.

For these deep sauropod casts, Xing et al. [59] suggested a more specific measurement and descriptive approach. However, QVI-SI1–4 is not fully exposed and thus some data are inaccessible. The upper surface of QVI-SI1 is about 55 cm in length and its lower surface is about 41 cm in length. QVI-SI1 has three clear digit traces in the anterior area, which are spaced by two grooves. The lateral digit is the largest and most likely to be digit I while the other two are likely digits II and III. The 23 cm deep digit area of QVI-SI1 is shallow, while the heel is 28 cm in depth. Obvious concave deformation can be seen below the track, forming a 14 cm deep undertrack area. Vertical to the track (especially digit I area) are several grooves and invertebrates trace casts. The former are spaced about 2–3cm apart and are probably traces made by the polygonal skin texture of sauropods which typically consists of an integument with a tightly-packed tubercle-like mosaic of polygons each up to 2–3 cm in diameter. Similarly wide striation traces have been observed in association with sauropod tracks at other localities [82]. The filled invertebrate traces suggest that, after the track was left, invertebrates lived or foraged in the depressions.

QVI-SI2 is behind QVI-SI1 and is oval shaped. Its upper surface is about 29 cm in length and its lower surface is about 24 cm in length and 17cm in depth. Based on position and size, QVI-SI2 is probably a manus track, and belonging to the same trackway as QVI-SI1. The lower surface of QVI-SI2 is crossed by two large mud cracks (about 2.5 cm deep). It is worth noting that about nine to ten 1.8–2.5 cm wide grooves are distributed transversely on the flanks of the track cast, forming small angles (~10°) with the upper and lower surfaces, and are not aligning with the common grooves, which are more vertical to the casts. They may be formed by the trackmaker’s polygonal skin ornament when turning its feet transversely in the sediment. In addition, at least two invertebrates traces run vertically to the track.

QVI-SI3 and SI4 are incomplete. The former is about 19 cm deep, similar to QVI-SI1 in morphology and size, most likely to be a pes cast, and has an undertrack about 5 cm deep. Partially preserved, QVI-SI4 is about 23cm deep and has well-preserved striation marks (about 1–2 cm wide) vertical to the cast. A cast is lost between QVI-SI3 and SI2 but there is an undertrack area about 24 cm deep. The undertack area likely formed when the sediment was affected by another pes trace.

The sixth layer also has about six casts of ornithopod tracks. These ornithopod tracks are about 7 cm deep and are generally much shallower than the sauropod casts; they co-occur with developed mud cracks. The sauropod trackmakers likely left tracks on relatively wet substrate, then tracks and substrate dried, and large mud cracks formed before the ornithopod tracks were left. In future, more sauropod tracks are likely to be discovered at the base of the sandstone ledges, ribbons, fins, and within the mudstone beds of the Jiaguan Formation. Such tracks are particularly conspicuous due to their large size.

## Bird Tracks

**Fig 16:**
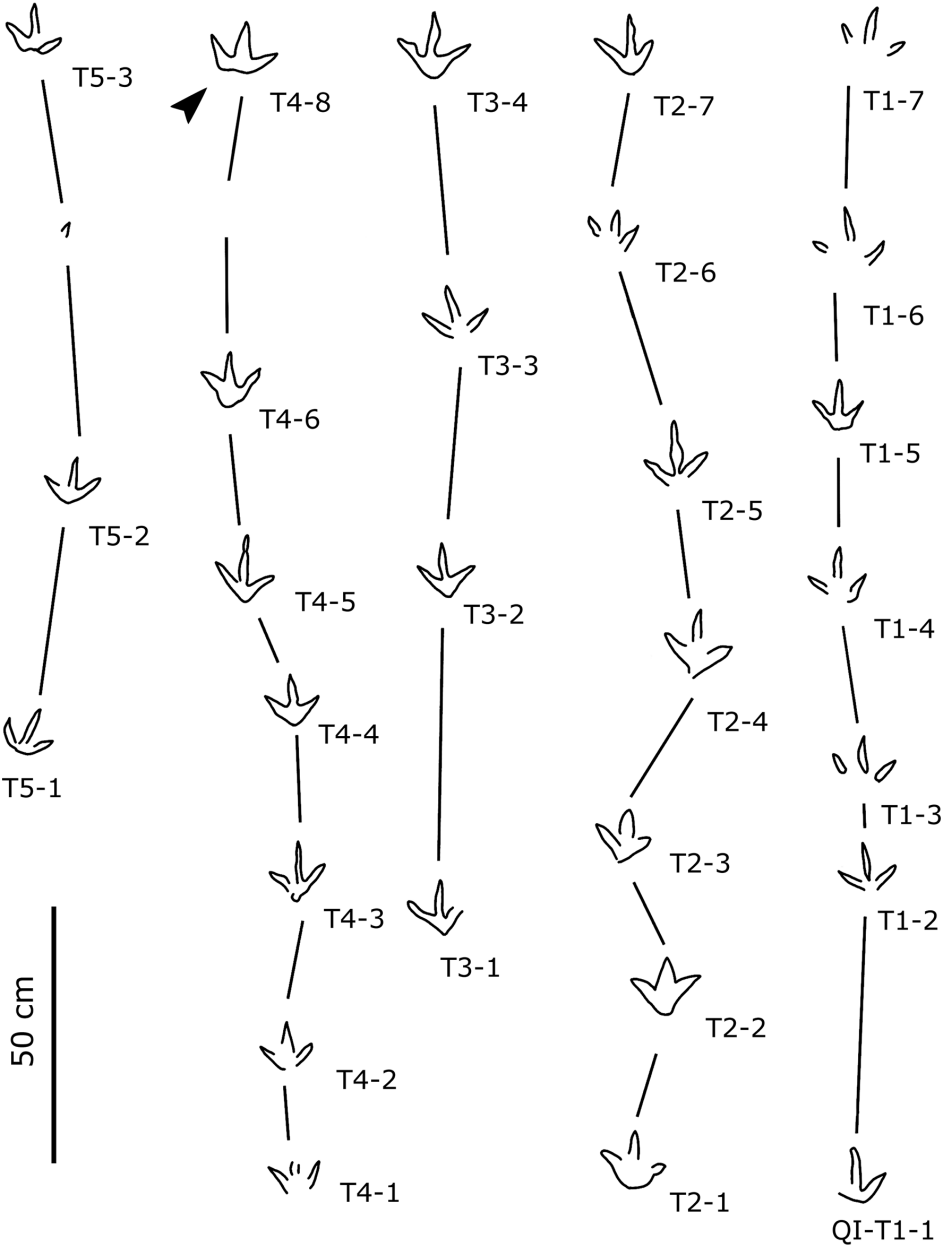
Interpretative outline drawing of bird trackways from the Lotus tracksite.

Trace fossils provide the only records of Early Cretaceous birds from many parts of the world. Previously, bird tracks from the Jiaguan Formation were rare. Specimens from the Emei tracksite were named *Aquatilavipes sinensis* [1], but *A*. *sinensis* was later reassigned to *Koreanaornis sinensis* [83]. *Wupus* from the Lotus tracksite, was originally identified as the trace of a small theropod track-maker [2] (Fig 16). It is similar in both footprint and trackway characteristics to the Early Cretaceous (Albian) large avian trace *Limiavipes curriei* [84] from western Canada, and *Wupus* is reassigned to the ichnofamily Limiavipedidae [4]. Reanalysis of *Wupus agilis* indicates that it represents the traces of a relatively large avian track-maker, analogous to extant herons. The analysis also reveals that, despite the current lack of body fossils, large wading birds were present globally in both Laurasia and Gondwana during the Early Cretaceous [4, 85–87]. A notable feature of the *Wupus* track assemblage on surface QI is that the trackways are nearly all parallel with an eastward orientation.

## Non-Avian Theropod Tracks

**Fig 17:**
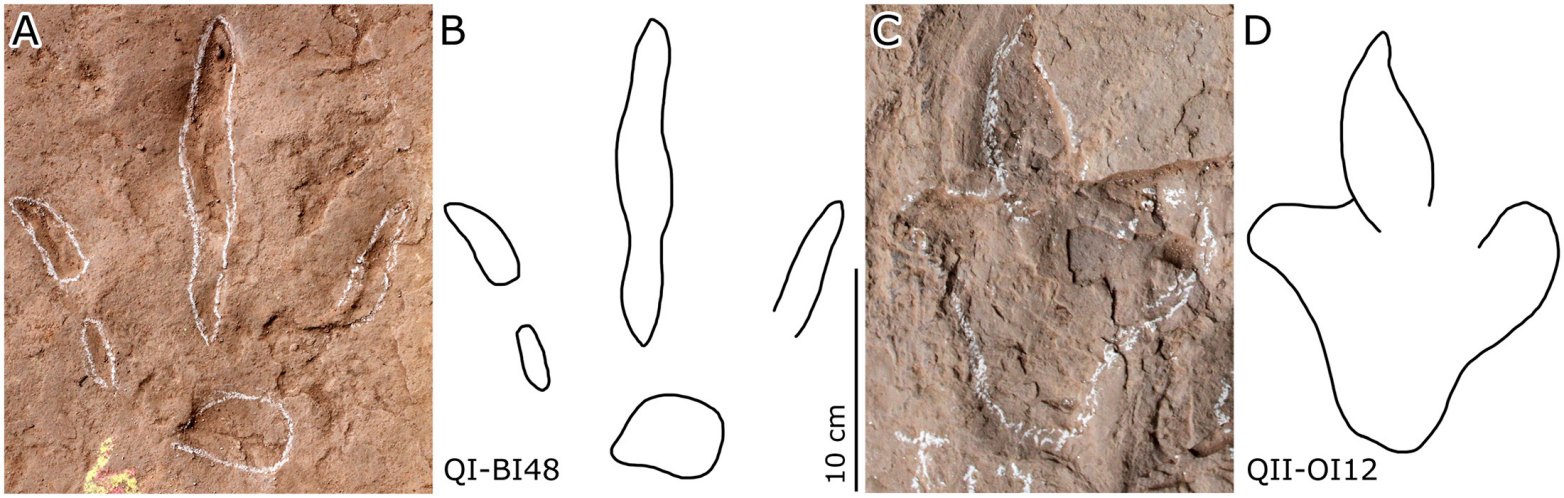
Photograph (A and C) and interpretative outline drawings (B and D) of possible theropod tracks from the Lotus tracksite.

Lotus tracksite lacks identifiable non-avian theropod tracks and only has two isolated tracks QI-BI48 and QII-OI12 (they were initially recognized as ornithopod undertracks and thus given a prefix of "O") from the first and second layer (Fig 17).

QI-BI48 is 20.7 cm in length, tridactyl, and has a L/W ratio of 1.16. It presents three slender digits with sharp claw marks at the distal ends, and an oval shaped metatarsophalangeal pad. Digit III has three digit pads, and the digit pad of the lateral digits is unclear. QI-BI48 co-occurs with *Wupus agilis* and is nearly identical to the latter in morphology, although it is much larger (~10 cm in *W*. *agilis*). QII-OI12 is a tridactyl track of 19 cm length and has a L/W ratio of 1.39. Digit III shows a sharp claw mark, while lateral digits are poorly preserved with indistinct digit pads. The developed metatarsophalangeal region is smoothly curved.

QI-BI48 and QII-OI12 meet morphological features of non-avian theropod track [88]. The mesaxony of QI-BI48 and QII-OI12 are 0.47 and 0.58, respectively, which is close to the footprints of the ichno- or morphofamily Eubrontidae [89]. Although QI-BI48 and QII-OI12 show affinity with non-avian theropod tracks, the small quantity and poor preservation complicates further comparison and discussion.

## Pterosaur Tracks

The Early Cretaceous was the height of pterosaur radiation. The number of known pterosaur tracksite reports from China has increased substantially in recent years. It compares favorably with the growing record from Korea and Japan, and adds significantly to the record from East Asia, such as Jimo, Shandong Province [90], Lotus, Chongqing Municipality [6], Xinjiang [91–92], Zhaojue, Sichuan Province [9], and Yanguoxia, Gansu Province [93].

The Lotus tracksite is one of the most important pterosaur tracksites in the Cretaceous of China due to the large number of imprints and their co-occurrence with bird tracks (*Wupus*). Xing et al. [6] described thirty tracks from five *Pteraichnus* trackways from the Lotus tracksite, and interpreted them as left by the same kind and similarly sized pterosaurs.

As in other *Pteraichnus* specimens from China, the *Pteraichnus* trackmaker from the Lotus tracksite was most likely a small to medium-sized pterodactyloid. However, a non-pterodactyloid trackmaker cannot be ruled out, because the impression of pedal digit V is rarely impressed clearly and unambiguously [94].

## Discussion

Tetrapod ichnofaunas from different layers at the Lotus tracksite vary in composition. Pterosaur and bird tracks were only registered on the first layer (QI), while ornithopod and sauropod tracks co-occur on some layers, but occur separately on others. Xing et al. [6] pointed out that small-sized theropods and pterosaurs probably preferred a stable sand bed, rather than highly-saturated muddy silt where they might sink in deep and expend more energy when walking [82]. After the second layer was covered by 10 cm of sandy sediment, a group of ornithopod track makers left tracks, some of which were transmitted to level QI as undertracks. Most tracks from the other layers are deep casts. This indicates the substrates represented by these layers were very soft.

If one trackway or one isolated track is regarded as reflecting an individual trackmaker, the abundance of trackmakers and proportions of different groups from the Lotus tracksite can be estimated (Table 7). Ornithopods dominate (69%) accounting for at least 165 trackmakers, followed by bird (18%), sauropod (10%), and pterosaur (3%). These proportions are similar to those at the Zhaojue tracksites, that are also located in the Sichuan-Yunan Basin. Ornithopods dominate (42%) in 76 trackmakers from Zhaojue tracksites, followed by theropods (25%), sauropods (24%) and pterosaurs (9%) [7–9]. However, Early Cretaceous ornithopod-dominated tracksites are unusual in China. Other important dinosaur-dominated footprint assemblages in China are mainly composed of saurischians, such as the theropod-dominated (~90%) Houzuoshan Dinosaur Park site from the Lower Cretaceous Tianjialou Formation at Junan County, Shandong Province [95]. The Yanguoxia tracksites are sauropod-dominated (38%), followed in abundance by theropods (32%), ornithopods (18%), birds (6%) and pterosaurs (3%) [58]. The Lower Cretaceous Jingchuan Formation represented at the Chabu sites in Inner Mongolia [96] lacks any confidently identified ornithopod tracks and is sauropod-theropod-bird dominated [56]. Besides, the Jehol Fauna, the most important and diversified Early Cretaceous dinosaur fauna in China [97], has produced countless specimens which are held in many institutions, therefore making a complete statistics nearly impossible. Therefore, based on only the Shandong Tianyu Museum of Nature which holds the biggest collection (about 3500 specimens of dinosaur-pterosaur material), ornithopods only account for 1%, while birds and non-ornithopod/theropod animals account for 65% and 19%, respectively. These differences may be explained in many ways, but most interpretations are necessarily speculative. In any event, the samples are very different in composition, in part due to sampling different sites, different regions and different facies all of which contribute certain biases to the fossil record. However, from the unusual ornithopod domination in the Qijiang track assemblage, in low latitude Early Cretaceous fluvial-lacustrine facies environments of the Sichuan-Yunan Basin, we can conclude that ornithopods were sometimes at least as commonas sauropods in these settings.

**Table 7:** Rank abundance of trackmakers by stratigraphic frequency of occurrence and total number of trackways (T) and isolated tracks (I).

	ornithopod		sauropod		bird		pterosaur	
	T	I	T	I	T	I	T	I
Lay I	—	—	—	—	5	175	5	—
Lay II	31	38	—	—	—	—	—	—
Lay III	7	17	1	9	—	—	—	—
Lay IV	—	2	—	—	—	—	—	—
Lay V	—	4	—	—	—	—	—	—
Lay VI	1	4	—	2	—	—	—	—
Lay VII	—	9	—	5	—	—	—	—
Total	39	74	1	16	5	25*	5	—

*175 isolated tracks comprise indistinguishable trackways. Based on five identified trackways, each one shows 7 tracks on average. Therefore, possible number of trackmakers can be estimated dividing isolated tracks (175) by 7.

## Conclusions

1. The *Caririchnium lotus* track assemblages associated with levels I, II and III are among the best-preserved and most significant of any known Cretaceous track assemblages, comprising at least 28 measurable trackways and an equal number of isolated specimens.
2. The trackways are morphologically similar to other *Caririchnium* ichnospecies from Brazil, North America and Korea, but differ in the configuration of the manus in most cases.
3. As is the case in most of these other regions the parallel trackways indicate gregarious behavior.
4. The assemblages indicate that at least two distinct cohorts were present indicating larger adults (Type A) and smaller sub adults (Type B). The adults all appear to have progressed quadrupedally, perhaps as obligate quadrupeds, whereas the smaller sub-adults progressed both as bipeds and quadrupeds: i.e., as facultative bipeds.
5. The *Caririchnium* assemblages are associated with bird- like tracks assigned to the ichnogenus *Wupus*, which is currently only known from this locality, and described in detail elsewhere [4], and the pterosaur tracks *Pteraichnus* also described in detail elsewhere [6]. Both these ichnogenera are confined to level QI.
6. Tracks named *Laoyingshanpus torridus* and *Qijiangpus sinensis* by Xing et al. [2] are here revaluated and regarded as undertracks transmitted from level II to level I. They are therefore considered extramorphological ichnites and here referred to as *nomina dubia*.
7. Casts of large sauropod tracks and ornithopod tracks occur at other levels (especially IV-VII).
8. Collectively the seven track-bearing levels indicate the presence of ornithopods, birds, pterosaurs and sauropods, with the former two groups being numerically dominant on the basis of raw trackway counts.
9. Collectively such ichnofaunas add vastly to our knowledge of the Cretaceous faunas known from the Jiaguan Formation and from the Lower Cretaceous of this region, which is otherwise very poorly represented by body fossils.

## Supporting Information

S1 Fig Map of track-bearing level at QI and II of the Lotus tracksite with trackway numbers. (PDF)

## Abbreviations

MWC: Museum of Western Colorado, U.S.A
SMU: Southern Methodist University, Texas, USA
QI–VII: Qijiang Layer I–VII, Lotus tracksite Chongqing Municipality, China
UCM: University of Colorado Museum of Natural History, USA
