# Supplementary figures and images for: An Ornithopod-Dominated Tracksite from the Lower Cretaceous Jiaguan Formation (Barremian–Albian) of Qijiang, South-Central China: New Discoveries, Ichnotaxonomy, Preservation and Palaeoecology
Source: PLoS One. 2015 Oct 22;10(10):e0141059. doi: 10.1371/journal.pone.0141059 (PMC4619635; doi:10.1371/journal.pone.0141059)

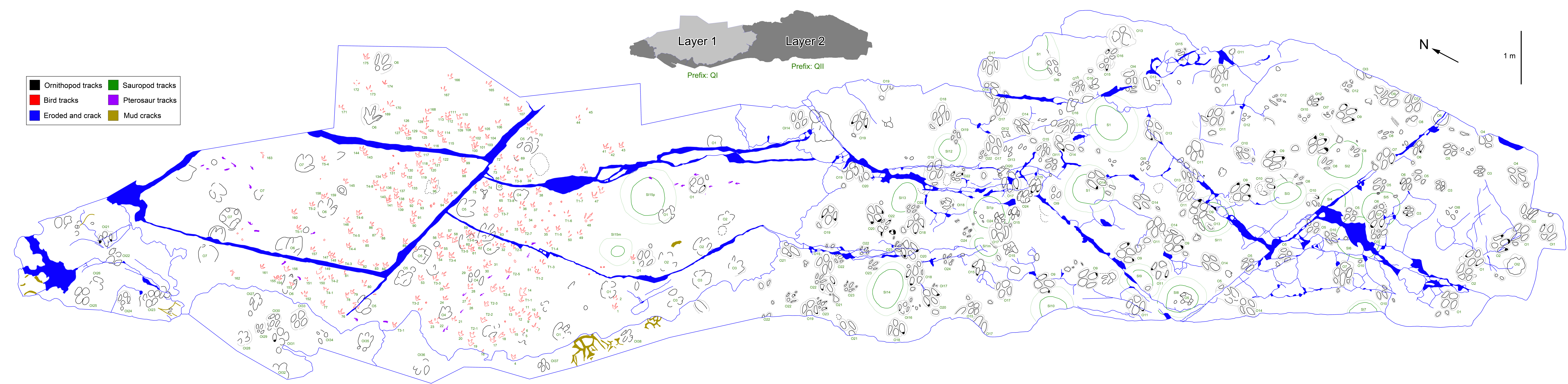

Supplement: S1 Fig — (PDF) [file pone.0141059.s001.pdf]
